# Inhibition of SHP2-mediated dephosphorylation of Ras suppresses oncogenesis

**DOI:** 10.1038/ncomms9859

**Published:** 2015-11-30

**Authors:** Severa Bunda, Kelly Burrell, Pardeep Heir, Lifan Zeng, Amir Alamsahebpour, Yoshihito Kano, Brian Raught, Zhong-Yin Zhang, Gelareh Zadeh, Michael Ohh

**Affiliations:** 1 Department of Laboratory Medicine and Pathobiology, University of Toronto, 1 King's College Circle, Toronto, M5S1A8 Ontario, Canada; 2 Brain Tumour Research Centre, Hospital for Sick Children, University Health Network, Toronto Medical Discovery Tower, 101 College Street, East Tower, Toronto, M5G1L7 Ontario, Canada; 3 Department of Biochemistry and Molecular Biology, School of Medicine, Indiana University, 635 Barnhill Drive, Indianapolis, Indiana 46202, USA; 4 Department of Biochemistry, University of Toronto, 1 King's College Circle, Toronto, M5S1A8 Ontario, Canada; 5 Princess Margaret Cancer Centre, Toronto Medical Discovery Tower, 9-701A, 101 College Street, Toronto, M5G1L7 Ontario, Canada

## Abstract

Ras is phosphorylated on a conserved tyrosine at position 32 within the switch I region via Src kinase. This phosphorylation inhibits the binding of effector Raf while promoting the engagement of GTPase-activating protein (GAP) and GTP hydrolysis. Here we identify SHP2 as the ubiquitously expressed tyrosine phosphatase that preferentially binds to and dephosphorylates Ras to increase its association with Raf and activate downstream proliferative Ras/ERK/MAPK signalling. In comparison to normal astrocytes, SHP2 activity is elevated in astrocytes isolated from glioblastoma multiforme (GBM)-prone H-Ras(12V) knock-in mice as well as in glioma cell lines and patient-derived GBM specimens exhibiting hyperactive Ras. Pharmacologic inhibition of SHP2 activity attenuates cell proliferation, soft-agar colony formation and orthotopic GBM growth in NOD/SCID mice and decelerates the progression of low-grade astrocytoma to GBM in a spontaneous transgenic glioma mouse model. These results identify SHP2 as a direct activator of Ras and a potential therapeutic target for cancers driven by a previously ‘undruggable' oncogenic or hyperactive Ras.

## Abstract (web summary)

Aberrant Ras signalling resulting in downstream Mek/Erk pathway activation is found in many cancers. Here, the authors show that the phosphatase SHP2 dephosphorylates Ras resulting in increased Ras activity, and that increased SHP2 activity is found in glioblastomas.

## Graphical abstract

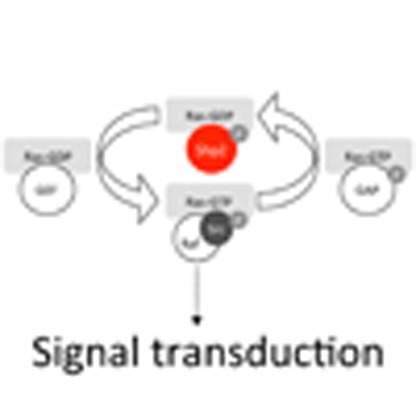

The three human Ras genes (*HRas*, *NRas* and *KRas*) are the most commonly mutated oncogenes in cancer. They encode highly related 188–189 amino-acid proteins that are membrane-bound GTP/GDP-binding proteins, which serve as a ‘molecular switch' linking receptor and non-receptor tyrosine kinase activation to downstream cytoplasmic and nuclear signalling events involved in a wide range of biological processes such as cell proliferation, differentiation and apoptosis^1^,^2^,^3^,^4^,^5^.

Ras cycles between the inactive GDP- and the active GTP-bound states^6^. Ras is turned ‘ON' by the exchange of GDP with GTP, catalysed by guanine-nucleotide exchange factors^7^. GTP-bound Ras has high affinity for effector molecules such as Raf, which activates the cell proliferative mitogen-activated protein kinase (MAPK) pathway^8^. Ras is subsequently turned ‘OFF' upon the hydrolysis of the bound GTP to GDP via its intrinsic GTPase activity, which is accelerated by GTPase-activating proteins (GAPs)^9^. Cancer-associated mutations in Ras, frequently in condons 12, 13 and 61, convert Ras into an active oncoprotein by impairing its GTPase activity^10^. Other components of the Ras signalling pathway have also been found to be altered in many cancers, which inappropriately activate the otherwise ‘normal' Ras to trigger various downstream signalling pathways^11^. For example, loss of or mutation in neurofibromin 1 (NF1 that encodes GAP) in neurofibromatosis type 1 (ref. 12), mutation in SHP2 (also known as PTPN11 that encodes protein tyrosine phosphatase) in juvenile myelomonocytic leukaemia^13^, and the overexpression of growth factor receptors in breast cancer^14^ and glioblastoma multiforme (GBM)^15^ have been associated with the hyperactivation of wild-type (WT) Ras.

GBM is the most common and most aggressive malignant brain tumour in humans. GBMs are classified as either primary or secondary GBM^16^. Primary GBM, which comprises ∼90% of all GBMs, develops rapidly *de novo*, whereas secondary GBM progresses from a low-grade astrocytoma to a high-grade glioma through the acquisition of additional genetic alterations^17^. In both cases, hyperactive receptor tyrosine kinases, epidermal growth factor receptor (EGFR) and platelet-derived growth factor receptor (PDGFR) potentiate common downstream Ras and phosphatidylinositol 3-kinase/Akt pathways to drive tumour growth and survival^18^,^19^. Thus, although mutations in Ras are rare in GBM, elevated levels of activated Ras are prevalently observed in GBM.

The ubiquitous tyrosine phosphatase SHP2 has emerged as a major regulator of the Ras/MAPK signalling pathway^20^. Germline-activating mutations in *SHP2* cause Noonan syndrome, whereas somatic gain of function *SHP2* mutations have been identified in several haematologic malignancies, most notably juvenile myelomonocytic leukaemia. However, the molecular mechanism by which SHP2 precisely activates the Ras/MAPK pathway remains unclear. Recently, we showed that Src-mediated phosphorylation of Ras promotes Raf displacement from Ras while enhancing GAP recruitment and subsequent GTP hydrolysis, thereby inactivating Ras^21^. Here, we show that SHP2 preferentially binds to and dephosphorylates tyrosyl phosphorylated Ras; an event that is required for the (re)activation of Ras and the continuation of Ras GTPase cycle. Notably, molecular or pharmacologic SHP2 inhibition attenuated the Ras activation and downstream MAPK signalling, and suppressed the progression of tumours in mouse models of GBM. We thus show that SHP2 is a direct activator of Ras and a potential therapeutic target for cancers driven by a previously ‘undruggable' oncogenic or hyperactive Ras.

## Results

### SHP2 dephosphorylates tyrosyl-phosphorylated Ras

**Figure 1:**
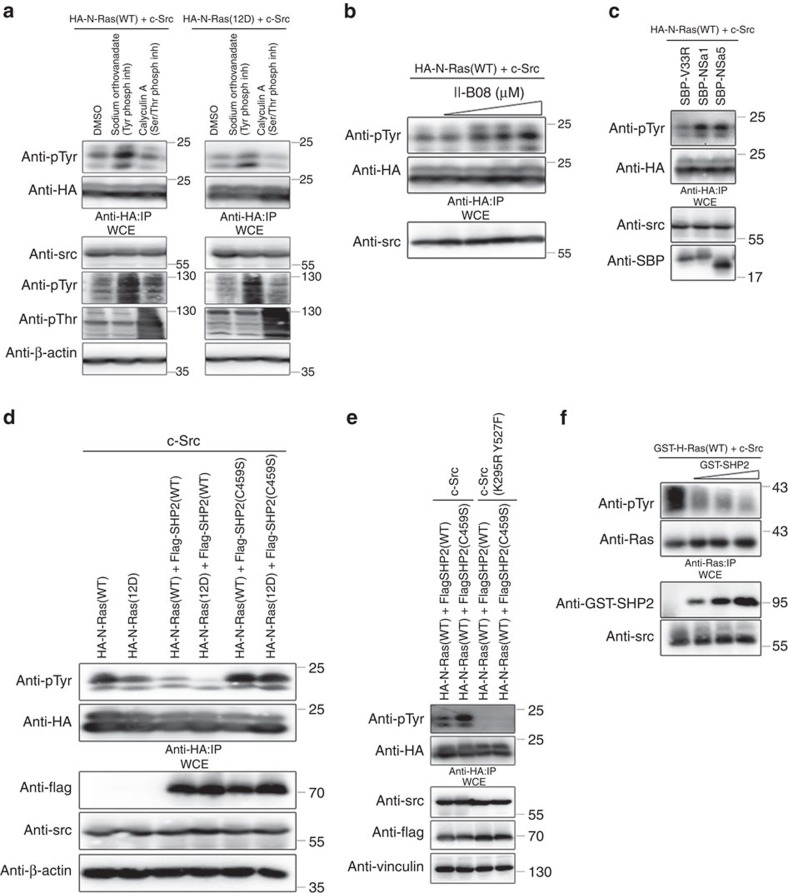
Tyrosine phosphatase SHP2 dephosphorylates wild-type and oncogenic Ras. (**a**) HEK293 cells transfected with the indicated plasmids were treated with the indicated inhibitors, lysed, immunoprecipitated with anti-HA antibody and immunoblotted with the indicated antibodies. (**b**) HEK293 cells transfected with the indicated plasmids were treated with increasing concentrations of II-B08, lysed, immunoprecipitated with anti-HA antibody and immunoblotted with the indicated antibodies. (**c**) HEK293 cells transfected with the indicated combination of plasmids and monobodies were lysed, immunoprecipitated with anti-HA antibody and immunoblotted with the indicated antibodies. (**d**,**e**) HEK293 cells transfected with the indicated plasmids were lysed, immunoprecipitated with anti-HA antibody and immunoblotted with the indicated antibodies. (**f**) Bacterially purified recombinant human GST-H-Ras(WT) was subjected to *in vitro* kinase assay in the presence of bacterially purified recombinant human active Src kinase. Subsequent dephosphorylation reaction was conducted using increasing amounts of bacterially purified recombinant human active GST-SHP2 followed by immunoprecipitation (IP) with anti-Ras antibody and immunoblotted with the indicated antibodies. The immunoblot data are representative of at least three separate experiments. WCE, whole-cell extract.

Recently, we showed that Src phosphorylates Ras on tyrosine 32 within the switch I region, which decreased the association between Raf and Ras while enhancing the binding of GAP to Ras to promote GTP hydrolysis and the inactivation of Ras^21^. Treatment of HEK293 cells co-expressing HA-N-Ras(WT or 12D) and c-Src with a general protein phosphotyrosine phosphatase inhibitor (sodium orthovanadate) increased the level of tyrosyl phosphorylated N-Ras(WT or 12D). By comparison, treatment with a serine/threonine phosphatase inhibitor calyculin A did not result in increased tyrosyl-phosphorylated N-Ras(WT) (Fig. 1a), suggesting that a tyrosine phosphatase actively dephosphorylates Ras.

Considering that a protein tyrosine phosphatase SHP2 has a pivotal role in the activation of the Ras/MAPK pathway^22^, we asked whether SHP2 is involved in the dephosphorylation of tyrosyl phosphorylated Ras. Pharmacologic inhibition of SHP2 using a specific cell-permeable SHP2 inhibitor, II-B08 (ref. 23), increased tyrosyl phosphorylated HA-N-Ras(WT) level in a dose-dependent manner (Fig. 1b). Treatment with another SHP2 inhibitor, PHPs1, or ectopic expression of two independent and highly specific and blocking SHP2 monobodies (NSa1 and NSa5 ref. 24), likewise increased HA-N-Ras(WT) phosphorylation in the presence of c-Src (Supplementary Fig. 1a and Fig. 1c, respectively). This effect was specific as it was not observed with the use of the V33R non-blocking control antibody. However, pharmacologic inhibition of another non-receptor phospho-tyrosine protein phosphatase, protein tyrosine phosphatase 1B had a negligible effect on HA-N-Ras(WT) phosphorylation (Supplementary Fig. 1b). Moreover, unlike WT SHP2, which attenuated HA-N-Ras(WT or 12D) tyrosyl phosphorylation in the presence of c-Src (Fig. 1d and Supplementary Fig. 1c), a catalytically dead SHP2(C459S) dominant-negative mutant allele or short hairpin RNA-mediated knockdown of endogenous SHP2 increased the level of tyrosyl phosphorylated HA-N-Ras(WT or 12D; Fig. 1d,e and Supplementary Fig. 1d). Notably, tyrosyl phosphorylation of HA-N-Ras was not observed in the presence of catalytically dead c-Src(K295R Y527F) mutant irrespective of the expression of WT or dominant-negative SHP2 (Fig. 1e). Recombinant GST-SHP2 dephosphorylated tyrosyl-phosphorylated recombinant GST-H-Ras(WT) in the presence of recombinant c-Src following an *in vitro* kinase assay (Fig. 1f). These results support the notion that SHP2 dephosphorylates c-Src-induced tyrosyl-phosphorylated Ras.

### SHP2 and Ras activities are elevated in GBM

**Figure 2:**
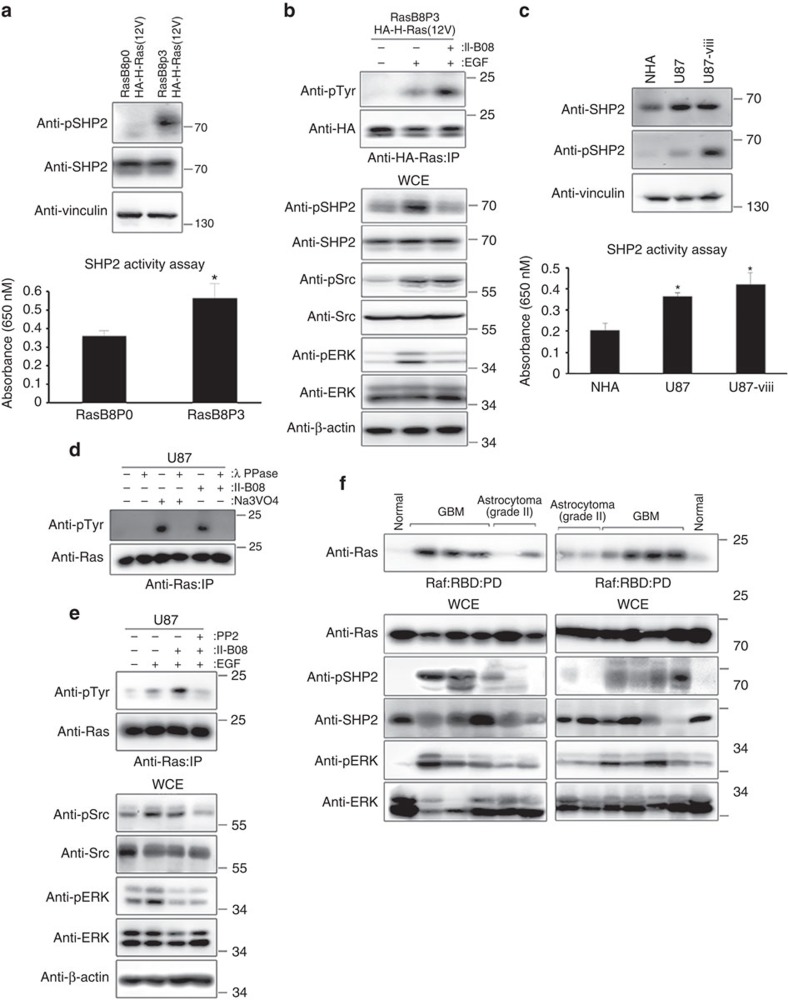
SHP2 and Ras activities are elevated in mouse and human GBM cell lines and primary patient-derived GBM specimen. (**a**) RasB8p0 or p3 astrocytes expressing the HA-H-Ras(12V) transgene derived from GBM-prone mice were either lysed and immunoblotted with the indicated antibodies (top panel) or subjected to SHP2 activity assay (bottom panel). Data represent mean±s.e.m. of three independent experiments performed in triplicate. *P<0.05 Student's *t*-test compared with RasB8p0. (**b**) RasB8p3 astrocytes were serum starved and pretreated with (+) or without (−) II-B08 followed by treatment with (+) or without (−) 1 ng ml^−1^ of EGF. Equal amounts of lysates were immunoprecipitated with anti-HA antibody and immunoblotted with the indicated antibodies. (**c**) Normal human astrocytes (NHA), U87 human GBM astrocytes or U87 astrocytes stably expressing EGFRviii deletion mutant (U87-viii) were either lysed and immunoblotted with the indicated antibodies (top panel) or subjected to SHP2 activity assay (bottom panel). Data represent mean±s.e.m. of three independent experiments performed in triplicate. *P<0.05 Student's *t*-test compared with NHA. (**d**) U87 astrocytes were treated with (+) or without (−) lambda phosphatase (λ PPase), II-B08 or sodium orthovanadate (Na_3_V0_4_) were lysed, immunoprecipitated with anti-Ras antibody and immunoblotted with indicated antibodies. (**e**) U87 astrocytes were serum starved and pretreated with (+) or without (−) PP2 or II-B08 and treated with (+) or without (−) 1 ng ml^−1^ of EGF, lysed, immunoprecipitated with anti-Ras antibody and immunoblotted with the indicated antibodies. (**f**) Patient-derived GBM or grade II astrocytoma samples were lysed pulled down using Raf:RBD beads, and immunoblotted with indicated antibodies. The immunoblot data are representative of at least three separate experiments. IP, immunoprecipitation; WCE, whole-cell extract.

Oncogenic HA-H-Ras(12V) knock-in RasB8 mice are prone to GBM^25^. Interestingly, astrocytes derived from newborn pups (RasB8p0) that are tumour free had markedly lower level of phosphorylated SHP2, which correlated with less SHP2 activity, compared with astrocytes derived from 3-month-old mice (RasB8p3) with GBM (Fig. 2a). Consistent with this observation, HA-H-Ras(12V) in RasB8p3 astrocytes had increased binding to Raf:RBD (Ras-binding domain) compared with RasB8p0 astrocytes (Supplementary Fig. 2a). Pharmacologic inhibition of the elevated SHP2 activity in RasB8p3 astrocytes using II-B08 or PHPs1 augmented epidermal growth factor (EGF)-induced tyrosyl phosphorylation of HA-H-Ras(12V) without affecting EGF-induced increase in Src phosphorylation, and attenuated the binding of Raf:RBD to HA-H-Ras(12V) (Fig. 2b and Supplementary Fig. 2b–d). These results suggest that the heightened SHP2 activity promotes oncogenic Ras(12V) dephosphorylation and activity in RasB8p3 astrocytes.

GBM cell line U87 harbours a hyperactive, but otherwise WT, Ras that is predominantly in the active GTP-bound form unlike normal human astrocytes (NHA), which harbour Ras that is predominantly in an inactive GDP-bound state^15^. U87-viii astrocytes associated with aggressive glioma cell proliferation express in addition a hyperactive EGFR variant III. SHP2 activity and phosphorylation level were significantly higher in U87-viii compared with U87 cells, which in turn show elevated SHP2 activity compared with NHA (Fig. 2c). II-B08 treatment of U87 cells attenuated SHP2 activity and markedly increased tyrosyl phosphorylation of Ras, which was abrogated in the presence of lambda phosphatase (Fig. 2d and Supplementary Fig. 2e,f). Notably, the enhanced tyrosyl phosphorylation of endogenous Ras observed in U87 and U87-viii astrocytes post-EGF and II-B08 treatment correlated with enhanced Src activity, which was markedly attenuated in the presence of Src kinase inhibitor PP2 (Fig. 2e and Supplementary Fig. 3a,b). These results further support the notion that SHP2 actively dephosphorylates Src-induced tyrosyl phosphorylated Ras.

We next examined SHP2 and Ras activity status in human primary GBM tumours, grade II gliomas and normal brain tissue, using tissue extracts assayed in a Raf-RBD buffer. GBM tumours showed the greatest elevation of Raf:RBD binding to Ras, whereas low-grade gliomas showed modest increase in Raf:RBD binding to Ras in comparison to normal brain tissue, which showed negligible binding of Ras to Raf:RBD (Fig. 2f). Notably, GBM tumours displayed markedly increased level of phosphorylated SHP2 and ERK followed by grade II gliomas in comparison to normal counterparts (Fig. 2f and Supplementary Fig. 4a). Elevated SHP2 phosphorylation status was also evident in four additional human GBM cell lines, U251, U118, A172 and T98G (Supplementary Fig. 4b), and SHP2 inhibition attenuated phosphorylated ERK and AKT levels in two different glioma stem cell (GSC) neurosphere cultures derived from fleshly operated tumour samples from GBM patients (Supplementary Fig. 4c). These results are consistent with the elevated SHP2 and Ras/pERK activity status observed in astrocytes derived from GBM-prone mice.

### Src-mediated phosphorylation of Ras promotes SHP2 binding

**Figure 3:**
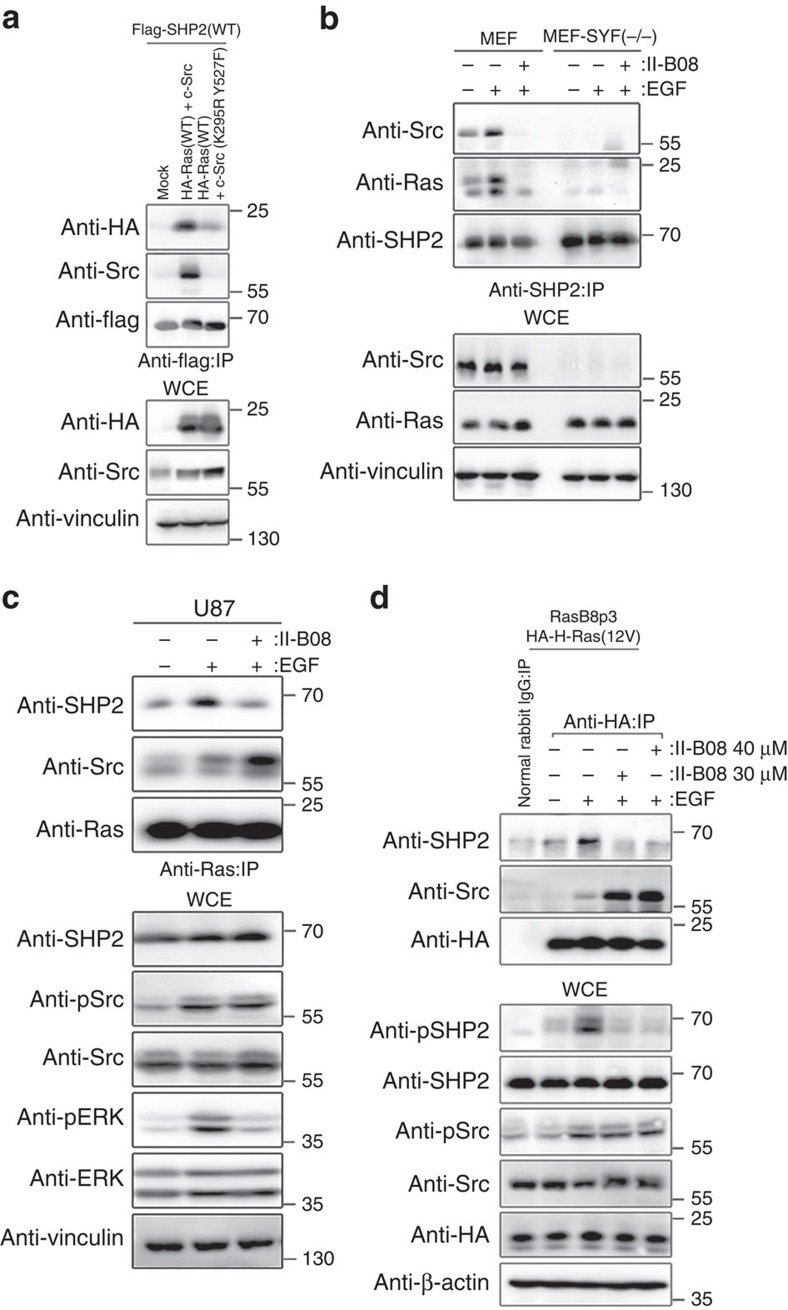
SHP2 binds to tyrosyl phosphorylated Ras in the presence of Src. (**a**) HEK293 cells transfected with the indicated plasmids were lysed, immunoprecipitated with anti-Flag antibody and immunoblotted with the indicated antibodies. (**b**) Wild-type MEFs or Src*/*Yes*/*Fyn triple-knockout (SYF−/−) MEFs were serum starved and pretreated with (+) or without (−) II-B08 and then treated with (+) or without (−) 1 ng ml^−1^ of EGF. Equal amounts of lysates were immunoprecipitated with anti-SHP2 antibody and immunoblotted with the indicated antibodies. (**c**) U87 astrocytes were serum starved and pretreated with (+) or without (−) II-B08 followed by a treatment with (+) or without (−) 1 ng ml^−1^ of EGF, lysed, immunoprecipitated with anti-Ras antibody and immunoblotted with indicated antibodies. (**d**) RasB8p3 astrocytes expressing the HA-H-Ras(12V) transgene were serum starved and pretreated with (+) or without (−) indicated concentration of II-B08 followed by treatment with (+) or without (−) 1 ng ml^−1^ of EGF, lysed, immunoprecipitated with control rabbit IgG or anti-HA antibody and immunoblotted with the indicated antibodies. The immunoblot data are representative of at least three separate experiments. IP, immunoprecipitation; WCE, whole-cell extract.

We asked whether c-Src mediated tyrosyl phosphorylation of Ras promoted SHP2 binding to Ras. Ectopically expressed HA-N-Ras(WT) co-precipitated with Flag-SHP2 and Src (Fig. 3a). Importantly, HA-N-Ras(WT) binding to Flag-SHP2 markedly increased when co-expressed with c-Src, but not in the presence of kinase-dead c-Src(K295R Y527F) (Fig. 3a and Supplementary Fig. 3c). In comparison to Flag-N-Ras(WT), Flag-N-Ras(Y32F) mutant that escapes tyrosyl phosphorylation by c-Src bound considerably less to Flag-SHP2(WT) (Supplementary Fig. 3d). These results suggest that c-Src-mediated phosphorylation of N-Ras promotes SHP2 recruitment to tyrosyl phosphorylated N-Ras. In support of this notion, endogenous Ras bound to SHP2 in WT mouse embryonic fibroblasts (MEFs), which was increased upon EGF treatment, but markedly attenuated in the presence of a cell-permeable SHP2 inhibitor II-B08 (Fig. 3b). Moreover, the interaction between Ras and SHP2 was negligible in Src/Yes/Fyn (SYF)-triple knockout MEFs (Fig. 3b). Notably, we have shown previously that EGF treatment fails to promote tyrosyl phosphorylation of Ras in SYF^−/−^ MEFs^21^. Furthermore, EGF-induced binding between SHP2 and Ras observed in U87 and RasB8p3 astrocytes was markedly diminished in the presence of II-B08 (Fig. 3c,d). These results suggest that SHP2 binding to Ras is dependent on the preceding Src-mediated phosphorylation of Ras.

**Figure 4:**
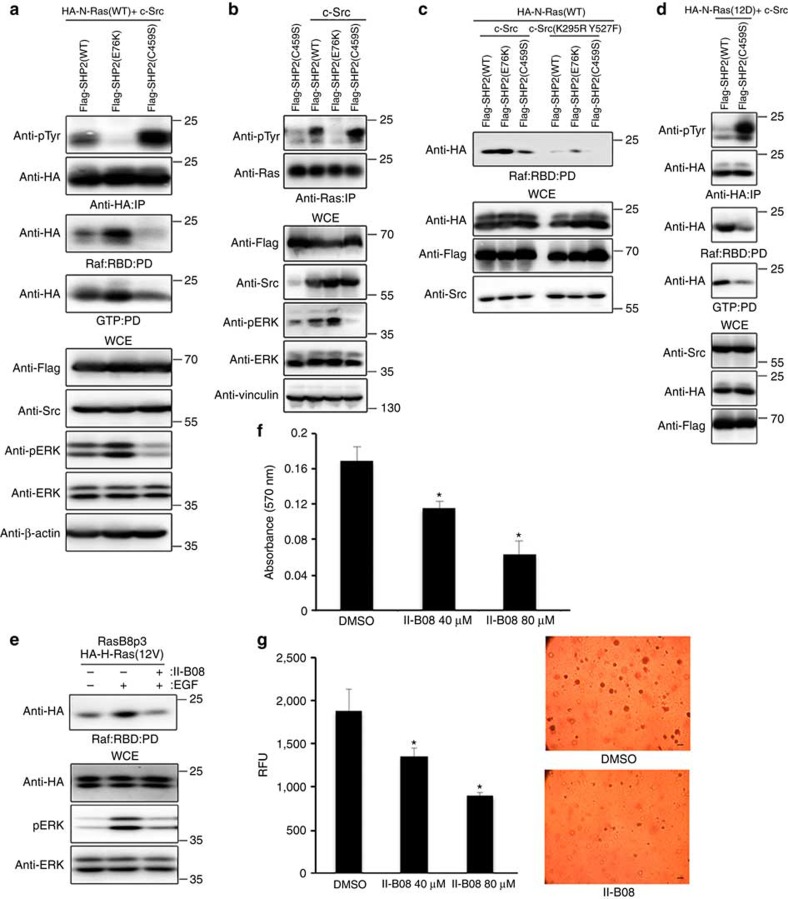
SHP2 inhibition promotes tyrosyl phosphorylation of wild-type and oncogenic Ras leading to inactivation of Ras and downstream signalling. (**a**) HEK293 cells transfected with the indicated plasmids were lysed, immunoprecipitated with anti-HA antibody or were pulled down using Raf:RBD beads or GTP beads, and immunoblotted with the indicated antibodies. (**b**) HEK293 cells transfected with the indicated plasmids were lysed, immunoprecipitated with anti-Ras antibody and immunoblotted with the indicated antibodies. (**c**) HEK293 cells transfected with the indicated plasmids were lysed, pulled down using Raf:RBD and immunoblotted with the indicated antibodies. (**d**) HEK293 cells transfected with the indicated plasmids were lysed, immunoprecipitated with anti-HA antibody or were pulled down using Raf:RBD beads or GTP beads, and immunoblotted with the indicated antibodies. (**e**) RasB8p3 astrocytes expressing the HA-H-Ras(12V) transgene were serum starved and pretreated with (+) or without (−) II-B08 followed by treatment with (+) or without (−) 1 ng ml^−1^ of EGF, lysed, pulled down using Raf:RBD beads and immunoblotted with indicated antibodies. (**f**) Equal number of RasB8p3 astrocytes were plated in 96-well plates in sextuplicate and treated with or without (dimethylsulphoxide (DMSO)) increasing concentration of II-B08 for 18 h and alamar blue assay was conducted according to the manufacturer's instructions. Data represent mean±s.e.m. of three independent experiments performed in sextuplicates. *P<0.05 Student's *t*-test compared with DMSO control. (**g**) Equal number of RasB8 astrocytes were suspended in agar matrix, and treated with or without increasing concentrations of II-B08. Following 10 days incubation, the anchorage-independent growth assay was performed according to the manufacturer's instructions. Data represent mean±s.e.m. of three independent experiments performed in sextuplicates. *P<0.05 Student's *t*-test compared with DMSO control. Representative images are also shown on the left panel captured using a Nikon Coolpix 995 digital camera mounted on a VWR Vista Vision inverted microscope. Scale bar, 200 μm. The immunoblot data are representative of at least three separate experiments. IP, immunoprecipitation; PD, pull down; WCE, whole-cell extract.

We next asked whether Ras activation is dependent on SHP2-dependent dephosphorylation of Ras. Compared with the WT SHP2, constitutively active disease-causing Flag-SHP2(E76K) mutant^26^ markedly reduced the level of tyrosyl phosphorylated N-Ras and increased the binding of N-Ras to effector Raf:RBD and GTP-conjugated agarose beads in HEK293 cells (Fig. 4a and Supplementary Fig. 5a). Conversely, the expression of catalytically inactive Flag-SHP2(C459S) mutant as well as endogenous SHP2 knockdown in HEK293 cells was associated with hyper-phosphorylation of N-Ras and reduced the binding of N-Ras to Raf:RBD and GTP-conjugated beads (Fig. 4a and Supplementary Fig. 5b,c). The enhanced level of tyrosyl phosphorylated ectopic or endogenous Ras in the presence of catalytically inactive Flag-SHP2(C459S) was also associated with reduced level of phosphorylated ERK, whereas the reduced level of tyrosyl phosphorylated Ras in the presence of constitutively active Flag-SHP2(E76K) was associated with increased level of phosphorylated ERK (Fig. 4a,b). Notably, the effect of SHP2(WT or E76K or C459S) on N-Ras binding to Raf:RBD was markedly muted in the presence of kinase-dead c-Src(K295R Y527F) mutant (Fig. 4c), which supports the notion that SHP2 regulates the activity of Ras via dephosphorylation of Src-phosphorylated Ras.

Notably, SHP2 inhibitor II-B08 treatment or ectopic expression of a dominant-negative Flag-SHP2(C459S) markedly enhanced phosphorylation of oncogenic HA-N-Ras(12D) mutant, concomitant with reduced binding to Raf:RBD and GTP-loaded beads and attenuated induction of EGF-induced phosphorylated ERK level (Fig. 4d and Supplementary Fig. 6). These results suggest that pharmacologic manipulation of SHP2 activity could influence the oncogenic potential of cancer-causing Ras mutants. In support of this notion, II-B08 treatment attenuated the elevated SHP2 activity and phosphorylation level observed in RasB8p3 astrocytes and enhanced tyrosyl phosphorylation of oncogenic HA-H-Ras(12V) following EGF treatment (Fig. 2b and Supplementary Fig. 2b). In addition, II-B08 treatment markedly suppressed the EGF-induced interaction between H-Ras(12V) and effector Raf:RBD and concomitantly decreased the level of phosphorylated ERK, cellular proliferation and growth of RasB8p3 astrocytes in soft agar (Fig. 4e–g, respectively). We took advantage of a spontaneous transgenic mouse model of human glioma, the RasB8 mouse model^25^, which is a model that forms low-grade gliomas that progress to a higher-grade glioma with malignant histopathological and molecular features in keeping with a GBM. Treatment of these mice with the Il-B08 drug results in decreased levels of phosphorylated SHP2 and phosphorylated ERK and a distinct lack of progression of the low-grade tumours to high-grade gliomas, as evidenced by a reduction in cellularity as determined by a decrease in nuclei and decrease in proliferation of astrocytic cells as determined on haematoxylin and eosin and astrocyte-specific marker glial fibrillary acidic protein (GFAP) (Supplementary Fig. 7).

**Figure 5:**
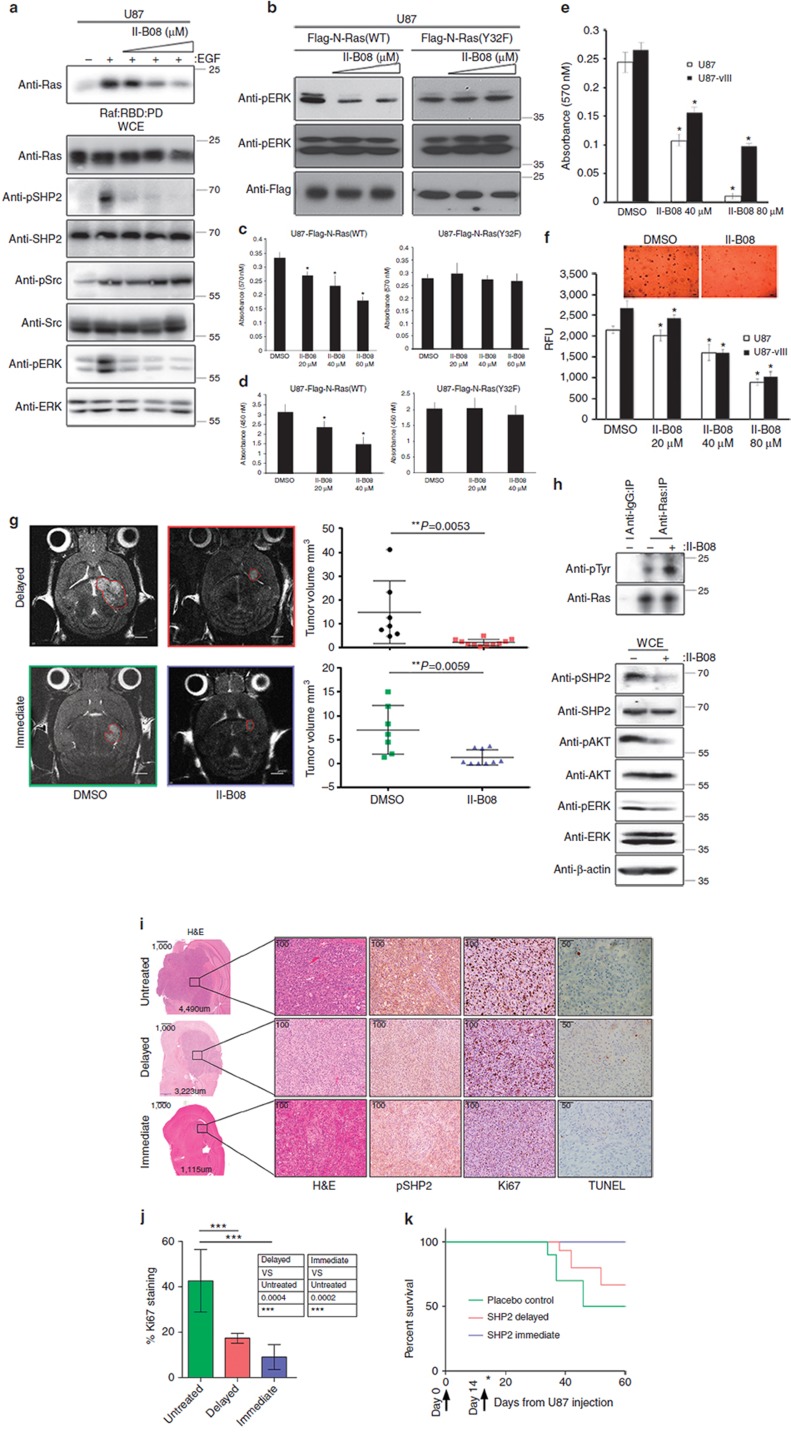
U87 xenograft mice treated with II-B08 demonstrate reduced tumour burden. (**a**) U87 astrocytes were serum starved and pretreated with (+) or without (−) increasing concentrations of II-B08 followed by treatment with (+) or without (−) EGF lysed and a pull down (PD) assay was conducted using Raf:RBD beads. (**b**) U87 astrocytes transfected with the indicated plasmids were treated with or without (dimethylsulphoxide (DMSO)) increasing concentration of II-B08 for 18 h, lysed and immunoblotted with the indicated antibodies. Equal number of U87 astrocytes transfected with indicated plasmids were plated in 96-well plates in sextuplicate, treated with or without (DMSO) increasing concentration of II-B08 for 18 h, and alamar blue (**c**) or 5-bromodeoxyuridine (BrdU; **d**) assay was then performed. Data represent mean±s.e.m. of three independent experiments performed in sextuplicates. *P<0.05 Student's *t*-test compared with DMSO control. (**e**) Equal number of U87 or U87 astrocytes stably expressing EGFRviii deletion mutant (U87-viii) were plated in 96-well plates in sextuplicate, treated with or without (DMSO) increasing concentration of II-B08 for 18 h, alamar blue assay was performed. Data represent mean±s.e.m. of three independent experiments performed in sextuplicates. *P<0.05 Student's *t*-test compared with DMSO control. (**f**) Equal number of U87 or U87-viii astrocytes were suspended in agar matrix and treated with or without increasing concentrations of II-B08. Following 10 days incubation, the anchorage-independent growth assay was performed. Data represent mean±s.e.m. of three independent experiments performed in sextuplicates. *P<0.05 Student's *t*-test compared with DMSO control. Representative images are shown on top panel captured using a Nikon Coolpix 995 digital camera mounted on a VWR Vista Vision inverted microscope. Scale bar, 200 μm. (**g**) T2 weighted anatomy MRI imaging demonstrated reduced tumour appearance after 7 days of therapy, which when quantified demonstrates a very significant decrease in the tumour volumetrics when compared with placebo (DMSO)-treated control mice following delayed therapy, beginning at 14 days post injection (**P = 0.0053, Student's *t*-test) and immediate therapy, beginning at the day of injection (**P = 0.0059, Student's *t*-test). Eigh*t*- to ten-week-old mice were used with *n* = 11 for delayed treatment group, *n* = 7 for delayed control group, *n* = 9 for immediate treatment group and *n* = 7 for immediate control group. Scale bar, 2 mm. (**h**) Right hemisphere containing the tumour was lysed from mouse treated with or without II-B08 and immunoprecipitated with anti-Ras or control IgG antibody and immunoblotted using the indicated antibodies. (*i*) Immunohistochemical staining of U87 tumour tissue following immediate and delayed Il-B08 treatments confirms a reduction in SHP2 and Ki67, whereas TdT-mediated dUTP nick end labelling (TUNEL), an apoptotic indicator, remained similar. Images are representative from untreated or immediate or delayed treated mice with II-B08. (j) Quantification of Ki67 as a % of total cells in the field demonstrates a statistically significant reduction following both treatments (***P<0.0001 ANOVA-post-Tukey). (k) Kaplan–Meier survival curve was plotted for control, immediate and delayed Il-B08-treated mice. The immunoblot data are representative of at least three separate experiments. IP, immunoprecipitation; WCE, whole-cell extract.

Consistent with the aforementioned RasB8p3 data, II-B08 treatment decreased the level of phosphorylated SHP2 and markedly attenuated the EGF-stimulated binding of Ras to Raf:RBD and phosphorylated level of ERK in a dose-dependent manner in U87 and U87-viii GBM cells, which harbour hyperactive, but otherwise WT, Ras (Fig. 5a and Supplementary Fig. 8). In addition, we showed previously that Ras is phosphorylated via Src on Y32 within the switch I region, which is critical for RasGTPase activity^21^. II-B08 treatment markedly reduced ERK phosphorylation, cellular viability and proliferation of U87 astrocytes expressing Flag-N-Ras(WT) but not of Flag-N-Ras(Y32F) phosphorylation-defective mutant expressing cells (Fig. 5b–d). Furthermore, ectopic expression of a dominant-negative Flag-SHP2(C459S) mutant markedly reduced Ras binding to Raf:RBD beads and attenuated phosphorylated ERK levels in Flag-N-Ras(WT)-expressing HEK293 cells, but had negligible effect on HEK293 cells expressing Flag-N-Ras(Y32F) mutant (Supplementary Fig. 9a). These results support the notion that SHP2 inhibition on proliferative signalling depends, at least in part, on the regulation of Ras phosphorylation status.

### Mice treated with II-B08 demonstrate reduced tumour burden

II-B08 treatment significantly reduced cellular proliferation and anchorage-independent growth of U87 and U87-viii astrocytes in a dose-dependent manner (Fig. 5e,f). U87-bearing xenograft mice when exposed to II-B08 demonstrated a significant reduction in overall tumour size 7 days after therapy was started when compared with those treated with placebo. This was demonstrated using T2weightd MRI imaging in both an immediate therapy group, beginning at the time of injection (**P=0.0059, Student's *t*-test), as well as in a delayed therapy group, beginning 14 days after injection (**P=0.0053; Fig. 5g). In comparison to U87 tumour-bearing brain isolated from untreated mice, Ras precipitated from II-B08-treated brain tissue showed markedly higher level of tyrosyl phosphorylated Ras concomitant with reduced level of phosphorylated SHP2 and downstream phosphorylated ERK and AKT (Fig. 5h). Immunohistochemical staining of tissue from the three groups confirmed smaller tumours in the treated mice, particularly the immediate group. Treated tumours had reduced SHP2 and Ki67, suggesting that treatment with II-B08 reduces the proliferative nature of the tumours and their overall growth (Fig. 5i). The reduction in proliferation is statistically significant in both the delayed and immediate treatment group (***P<0.0001, analysis of variance followed by post-Tukey; Fig. 5j). The reduced proliferative nature of the tumour is consistent with the observed trend of increased survival at 60 days in immediate and delayed II-B08 treatment groups in comparison to the placebo-treated control group (Fig. 5k).

## Discussion

SHP2 is the first identified proto-oncogene encoding a cytoplasmic tyrosine phosphatase that promotes the activation of the Ras/ERK/MAPK pathway in response to growth factors, cytokines and hormones^27^,^28^,^29^. SHP2 contains the obligatory protein tyrosine phosphatase domain, two tandem Src homology 2 (SH2) domains and several tyrosine residues that are targeted for phosphorylation. SH2 domains are necessary for SHP2 engagement on phosphotyrosine in a variety of growth factor receptors and signalling molecules^29^. However, although several mechanisms have been proposed, how SHP2 precisely regulates Ras/ERK/MAPK pathway is unclear. We recently showed that Src kinase binds to and preferentially tyrosyl-phosphorylates GTP-loaded Ras on tyrosine at position 32 within the switch I region; an event that is associated with Raf displacement while increasing GAP binding and GTP hydrolysis^21^. Here, we show that tyrosyl-phosphorylated Ras binds to and is dephosphorylated by SHP2, which in turn promotes Ras binding to Raf and the activation of downstream Ras/ERK/MAPK signalling. These results support the notion that SHP2 reactivates the inactive tyrosyl-phosphorylated Ras via dephosphorylation to begin a new Ras GTPase cycle.

An efficient SHP2 substrate generally contains two or more acidic residues on the N-terminal side and one or more acidic residues on the C-terminal side of phosphorylated tyrosine (pY) with no basic residues^30^. SHP2 also prefers the acidic residue aspartic acid (D) at pY −2 position. The sequence surrounding Ras-pY32, FVDEpY(32)DPTIED, is consistent with the general SHP2-binding consensus motif. Notably, co-operation between c-Src-mediated tyrosyl phosphorylation of a target protein and subsequent dephosphorylation by SHP2 has been shown recently for the transcription factor RUNX1. Similar to our findings, Src-mediated tyrosyl phosphorylation of RUNX1 negatively affected RUNX1 activity, whereas SHP2-dependent dephosphorylation of RUNX1 rescued the negative regulation via Src^31^. It is formally unknown whether SHP2-dependent dephosphorylation of other signalling molecules such as Alpha7 neuronal nicotinic acetylcholine receptors would rescue the negative effect of phosphorylation induced by Src family kinases^32^.

SHP2 has both phosphatase-dependent and -independent functions via its ability to bind to various signalling molecules^33^. For example, it is well established that tyrosyl phosphorylated SHP2 binds to the adapter Grb2 (ref. 34). Grb2 binds to the guanine nucleotide exchange factor Son of Sevenless (SOS), which promotes the removal of GDP from Ras for GTP, a necessary step in the re-activation of Ras^35^. We postulate that following SHP2 binding and dephosphorylation of Ras, which requires SHP2's phosphatase function, SHP2 via its adapter function recruits Grb2/SOS complex directly onto Ras to promote Ras activation and subsequent Raf engagement. Although formally unknown, it is thus likely that SHP2-mediated activation of Ras/MAPK signalling requires both SHP2 phosphatase activity as well as its adapter function.

SHP2 phosphatase function has also been shown to inhibit recruitment of RasGAPs to the cell membrane^36^. We recently showed that tyrosyl phosphorylation of Ras promotes binding to RasGAP and GTP hydrolysis^21^. Thus, SHP2-dependent Ras dephosphorylation would hinder RasGAP binding to Ras or the recruitment to the cell membrane as noted above; both of which would increase or prolong Ras activation. SHP2 has also been shown to dephosphorylate the negative Ras regulator Sprouty to activate the Ras/ERK/MAPK signalling pathway in the presence of WT^37^ but not oncogenic Ras^38^. However, we show here that the catalytically inactive SHP2 mutant attenuates oncogenic Ras activation, which questions the involvement of Sprouty in SHP2-mediated regulation of Ras. Moreover, Sprouty has also been shown to negatively or positively regulate RTK signalling depending on interacting partners and cell context^39^. SHP2 has been suggested to activate Ras/MAPK signalling via the activation of Src^40^. However, Src on its own does not fully activate but rather partially contributes to the activation of Ras/MAPK signalling by phosphorylating Raf alongside other serine threonine kinases^41^. We show here that pharmacological or molecular inhibition of SHP2 phosphatase function does not lead to the inactivation of Src, but rather promotes Ras phosphorylation in the presence of catalytically active Src.

Gain-of-function mutations in the SHP2 gene, *PTPN11,* have been identified in Noonan Syndrome^42^, haematological malignancies^43^,^44^,^45^,^46^,^47^ and other types of cancers, including breast cancer^48^, lung cancer, neuroblastoma, pilocytic astrocytoma and medulloblastoma^49^,^50^,^51^,^52^,^53^,^54^. According to the Cancer Genome Atlas database, ∼2% of GBM patients harbour *PTPN11* mutations^55^. Despite the rarity of *PTPN11* mutations in gliomas^53^,^56^, other components of the Ras signalling pathway, such EGFR, NF-1 and Ras, are genetically altered or deregulated in the majority of GBM^57^. Here, we revealed the molecular mechanism by which SHP2 regulates Ras activity and show that elevated SHP2 activity drives GBM progression via Ras activation. Inhibiting SHP2 activity with II-B08 led to the inactivation of this difficult to target oncogene by promoting its phosphorylation and inhibiting Raf binding. As Ras transduces signals from many different receptor tyrosine kinases that are known to be overexpressed in GBM, including EGFR, FGFR and PDGFR, targeting SHP2 may prove more effective in GBM, which respond poorly to kinase inhibitor monotherapy.

We tested the impact of inhibiting SHP2 in a tumour model where Ras activity is relevant. We took advantage of a spontaneous transgenic RasB8 mouse model of malignant glioma, in addition to using orthotopic intracranial xenograft models. Following SHP2 inhibition with the administration of II-B08, there was a clear prevent of progression to higher-grade gliomas, with histopathological features of malignant gliomas not evident in the treated animals. In addition, there were reduced levels of Ki67 proliferation and an overall reduction in tumour cell density as determined by nuclei in the haematoxylin and eosin-stained tissues. Given the diffuse and random nature of the gliomas that form in these mice, measuring overall volume is not an accurate or meaningful end point. To determine whether SHP2 inhibition results in decrease tumour growth, we used an orthotopic model of malignant glioma by injecting malignant human glioma U87 cells intracranially in NOD/SCID mice. When compared with control mice, SHP2 inhibition resulted in a reduction in tumour growth and decreased Ki67 proliferation, which were associated with increased survival of II-B08-treated mice at 60 days post implantation of malignant U87 glioma cells.

As confirmed by the *in vitro* work, the inhibition of SHP2 is able to perturb the RasB8 signalling pathways resulting in elevated levels of Ras in its phosphorylated state. Phosphorylated Ras has a lower affinity for its effector Raf that in turn leads to a reduction in activated downstream proliferative MAPK/ERK signalling. Overactive Ras levels are a common precursor in both secondary and primary GBMs and most notably there is an increase in overactive aberrant Ras in high-grade gliomas when compared with both low-grade and normal brain. Furthermore, SHP2 has been shown to be an important player in the classical subgroup of GBM but not in other less tumourigenic subgroups as defined by the Cancer Genome Atlas. SHP2 also mediates PDGFRα-induced gliomagenesis and tumour invasion^58^,^59^ and contributes to EGFRviii-induced cell transformation and tumour growth^22^,^60^. It is therefore plausible that targeting Ras signalling via the inhibition of SHP2 would attenuate the growth of gliomas in humans and, perhaps equally important, block the progression of lower grade gliomas to a higher grade.

## Methods

### Cells

HEK293, MEF, MEF-SYF(−/−) and U87, U251, U118, A172 and T98G were obtained from American Type Culture Collection. NHA, U87-vIII cell line, an EGFRviii expression derivative of U87, RasB8 p0 and p3 cells were generated as previously described^25^. HEK293-shSHP2 cells were generated using transduction-ready SHP2 or control non-targeting short hairpin RNA lentiviral particles according to the manufacturer's instructions (Santa Cruz Biotechnology) and stably expressing cells were isolated by puromycin selection. GSCs were derived from freshly operated tumour samples from GBM patients at the University of Texas MD Anderson Cancer Center as per guidelines set by institutional review board guidelines. Each patient provided written informed consent for tumour tissues and this study was conducted under protocol LAB03-0687, which was approved by the institutional review board of the University of Texas M. D. Anderson Cancer Center as described previously^61^. HEK293, MEF and MEF-SYF(−/−) cells as well as U87, U87-viii, NHA, U251, U118, A172, T98G, RasB8 p(0) and p(3) cells were maintained in DMEM (Invitrogen) supplemented with 10% heat-inactivated fetal bovine serum (Wisent) at 37 °C in a humidified 5% CO_2_ atmosphere. GSCs were maintained as neurospheres in defined DMEM/F12 media in the presence of growth factors EGF (20 ng ml^−1^), recombinant basic fibroblast growth factor (bFGF) (20 ng ml^−1^; R&D systems) and B27 growth supplement with vitamin A (1:50 working concentration; Life Technologies).

### Plasmids and monobodies

Plasmids encoding human pCGN-HA-N-Ras(WT or 12D), pCMV5-c-Src(WT or K295R) and pCMV5-SHP2(WT) were obtained from Addgene. Flag-SHP2 constructs were obtained from Addgene and subcloned into pcDNA3. Plasmids were verified by direct DNA sequencing. Monobodies directed against SHP2 were generated as previously described^24^ and generously provided by Dr Shohei Koide.

### Xenograft models of GBM

All animal procedures were carried out according to animal user protocols approved ethically by the Institutional Animal Care Committee under the guidelines of the Canadian Council on Animal Care and the University Health Network Research Ethics Board. Chimeric BM mice (male 8 weeks) were anaesthetized using 0.5 mg g^−1^ of intraperitoneal injection of 20 mg ml^−1^ Avertin (Sigma-Aldrich) and 5 mg kg^−1^ of the pre-surgical analgesic Anafen 1 mg ml^−1^ (Ketoprofen) was administered subcutaneously. TearGel (Novartis) was applied to the eyes to prevent corneal dehydration and abrasion. Once a toe pinch no longer elicited a response, the scalp was cleaned and hair removed, and a midline incision was made from the ears to the eyes. Underlying periosteium was frozen with 2% Lidocaine (Bimeda MTC) and removed with scissors. Mice were placed on a digital stereotaxic frame and from bregma the cortex coordinates were identified (X:1.6, Y:1, Z:0.6). A high-speed dental drill with a 0.7-mm adaptor (Fine Science Tools) was used to bore a whole through the skull. 2 × 10^5^ U87 cells resuspended in 10 μl of sterile 1 × PBS were injected 3 mm deep through the hole using a 10-μl 30-G Hamilton microsyringe, over a 1-min time period. Control mice were sham injected. Mice were sutured and returned to a fresh sterile heated cage to recover and supplemented with 0.3 mg ml^−1^ Enrofloxacin in the drinking water (Baytril Bayer/CDMV, cat. no.102207).

### Transgenic mouse models

GBM transgenic mouse model, RasB8, was generated through integration of a V12 RasB8 mutation under the control of GFAP-promoter leaving mice predisposed to sporadic GBM-like astrocytoma^25^. Embryonic stem cell complementation methodology was used to integrate a V12 RasB8 mutation (IRES LACz) under the control of GFAP-promoter into an ICR background strain mouse. The positive RasB8 male mice are bred heterozygously to ICR females, as the homozygous crosses are lethal before P14 (ref. 25). Genotyping is carried out for both the RasB8 mutation and the LACz reporter construct; in addition, LACz immunohistochemistry (IHC) is carried out to ensure full protein translation.

### MRI

A 7-Tesla Bruker model BioSpec 70/30 MR system with B-GA12 gradient coil, 7.2 cm diameter linear radiofrequency transmitter coil, murine head radiofrequency receiver coil and murine slider bed was used for serial imaging. Mice under isoflurane anaesthesia were positioned on the MR bed with a bite block and water warming system to maintain body temperature during imaging. Serial multiparametric MRI protocol was carried out as previously described^62^.

### Patient-derived tumour samples

Patient resected samples were obtained from Toronto Western Brain Tumour Bank and processed in accordance with a University Health Network Research Ethics Board-approved protocol.

### Antibodies and recombinant proteins

Purified recombinant GST-H-Ras(WT) was obtained from Jena Bioscience. Active recombinant human His-c-Src kinase and GST-SHP2 were obtained from GenWay Biotechnology. Rabbit polyclonal antibodies against Src, phosphorylated (p)Src(Y416), pAKT, AKT, glutathione *S*-transferase (GST), pSHP2(Y542), SHP2 and HA (polyclonal) were obtained from Cell Signaling Technologies. Polyclonal IgG (sc-2027), pERK (sc-7383), N-Ras (sc-519), and c-Raf (sc-7267) were obtained from Santa Cruz Biotechnology. p-c-Raf(Y340) (#44506G) was obtained from Invitrogen. GFAP and Ki67 were obtained from Dako. Monoclonal antibodies against HA (12CA5) and pTyr (4G10) (05-321) were obtained from Boehringer Ingelheim and Millipore, respectively. Monoclonal FLAG-M2 (F1804), β-actin (A5316) (1:10,000), vinculin (V9264) (1:10,000) and polyclonal ERK (M5670) antibodies (1:5,000) were obtained from Sigma. All antibodies were utilized at a 1:1,000 dilution unless otherwise specified.

### Chemicals

To prepare SHP2 inhibitor II-B08, the critical intermediate methyl 3-ethynyl-6-hydroxy-1-methyl-2-phenyl-1H-indole-5-carboxylate was prepared as previously described^23^ Catalysed by Tetrakis(acetonitrile)copper(I) hexafluorophosphate in dimethylformamide (DMF), the above intermediate compound was reacted with *N*-([1,1'-biphenyl]-4-yl)-3-azidopropanamide and then hydrolysed to yield II-B08 (two steps yield 64%) in a mixed solvent of tetrahydrofuran (THF), methanol and 5% LiOH solution (volume ratio: 10:2:3). PHPS1 was obtained from Millipore. Protein tyrosine phosphatase 1B was obtained from Calbiochem. PP2 and calyculin A were obtained from Sigma. EGF was obtained from Invitrogen. Ready to use sodium orthovanadate was obtained from FIVEphoton Biochemicals. Lambda phosphatase was obtained from New England Biolabs. Immobilized GTP-agarose was obtained from Jena Bioscience.

### Immunoprecipitation and immunoblotting

Immunoprecipitation and western blotting were performed as described previously^63^. Cells were harvested in EBC lysis buffer (50 mM Tris, pH 8, 120 mM NaCl, 0.5% NP-40) and supplemented with protease inhibitors (Roche). Lysates were immunoprecipitated using the indicated antibodies along with protein A-Sepharose (Repligen). In experiments assessing active Src, cell lysates were incubated with anti-v-Src (Ab-1) mouse (327) agarose-conjugated antibody (Millipore). Bound proteins were washed five times in NETN buffer (20 mM Tris, pH 8, 100 mM NaCl, 1 mM EDTA, 0.5% NP-40), eluted by boiling in sample buffer and resolved by SDS–polyacrylamide gel electrophoresis. Proteins were electro-transferred onto polyvinylidene difluoride membrane (Bio-Rad), blocked and probed with the indicated antibodies. All uncropped western blots are presented in Supplementary Fig. 10.

### Ras activity assay

Ras activity was assessed using the Ras activation assay kit from Millipore. Briefly, Ras-GTP from various treated lysates was pulled-down using the GST fusion protein corresponding to human Ras-binding domain of Raf-1 bound to agarose. The presence of Ras-GTP was detected by western blotting using anti-Ras antibody (Millipore).

### *In vitro* kinase and SHP2 dephosphorylation assay

Bacterially purified recombinant human GST-H-Ras(WT) was combined with bacterially purified recombinant human active His-c-Src kinase in 100 μl of vitro kinase buffer (50 mM HEPES (pH 7.5), 10 mM MgCl_2_, 1 mM EGTA, 0.01% Brij-35, 200 μM ATP) 1 h at room temperature. Thereafter, 1 ml of binding buffer was added along with glutathione-Sepharose. Bound proteins were washed five times in binding buffer and final wash was conducted in SHP2 dephosphorylation buffer and then incubated at 30 °C for 30 min with increasing amount of recombinant active GST-SHP2 as per manufacturer's instructions. Bound proteins were washed five times in binding buffer before elution by boiling in sample buffer.

### SHP2 activity assay

SHP2 phosphatase activity of treated or untreated cell lysates with II-B08 was determined using the human/mouse/rat active DuoSet IC kit (R&D Systems), according to the manufacturer's instructions. Briefly, the total cellular SHP2 bound to anti-SHP2 antibody conjugated to agarose beads was exposed to synthetic phosphopeptide substrate, which is only dephosphorylated by active SHP2 to generate free phosphate and unphosphorylated peptide. The amount of free phosphate in the supernatant was determined by a sensitive dye-binding assay using malachite green and molybdic acid and represents a direct measurement of SHP2 activity in the cellular system.

### Cell proliferation assay

Equal numbers of cells were plated in quadruplicate in 96-well plates in the presence or absence of indicated inhibitors and cellular proliferation was assessed using Alamar Blue proliferation assay as per manufacturer's instructions (Invitrogen) or 5-bromodeoxyuridine cell proliferation assay as per manufacturer's instructions (BioVision).

### Anchorage-independent growth assay

Anchorage-independent transformation assays were performed using a CytoSelect 96-well cell transformation assay kit (Cell Biolabs, Inc.). Briefly, equal number of cells were plated in soft agar in a 96-well plate and cultured in the presence or absence of indicated inhibitors. The transformation was determined according to the protocol provided by the manufacturer.

### Immunohistology

Paraffin sections of intracranial injected U87 orthotopic xenografts and RasB8 mice brains were de-paraffinated overnight with xylene then rehydrated through 100, 95, 70% and ddH_2_O. Antigen pretreatment in low pH buffer (Vector labs H330) was carried out with boiling under pressure for 3 min. Next endogenous peroxidase was inhibited using 0.3% hydrogen peroxide in methanol for 20 min. 10% serum derived from the secondary antibody source was used to block for 30 min. Sections were incubated for 1 h at room temperature with indicated primary antibodies. Biotinylated secondary antibodies (Vector Labs) were next applied for 1 h at room temperature. IHC stains underwent a 30-min horseradish peroxidase-conjugated ultrastreptavidin labelling (ID labs) treatment and colour was developed using freshly prepared DAB solution (Vector Labs), slides were lightly counterstained with haematoxylin, dehydrated in alcohols, cleared in xylene and mounted in Permount (Fisher). For TdT-mediated dUTP nick end labelling stain, rehydrated slides were placed in ice-cold Alcohol/Acetic acid mixture (2:1) for 5 min then rinsed with PBS. Sections were treated with buffer A (50 mM Tris-HCl, pH 7.5, 50 mM MgCl_2_, 100 mM β-Mercaptoethanol, 0.005% BSA) for 5–10 min before being incubated in a biotin nucleotide cocktail at 37 °C for 90 min. After washing in PBS, Alkaline Phosphatase Strepavidin Reagent (Vector Labs, SA-5100) was applied for 30 min. Colour was then developed using freshly prepared DAB before being lightly counterstained with Mayer's haematoxylin and mounted with Permount.

### Image analysis

For IHC data quantification, ten high-powered fields from minimum five samples were blindly analysed using Image-J software. Briefly, RGB images were split and converted to 8 bit gray-scale images. Threshold levels were adjusted (45pi< × <225pi) to mask nuclei, resulting images were converted to binary for analysis. Particle analysis was completed based upon size restrictions of 8pi^2^–infinity leaving morphology unspecified. Intensity thresholds were then modified in the same way to account for positively stained cells. MRI images were analysed as a stack using MiPAV software. Tumour areas were defined as ROI by three-blinded personnel on each image and a reconstructed volumetric algorithm was setup to define total volume. Averages were taken for each sample.

### Statistical analysis

All experiments were performed in triplicate with mean and standard error reported. Analysis of variance) followed by a post-Tukey for pairwise comparisons. For direct comparisons, an unpaired Student's *t*-test was carried out. Significance was defined as *P<0.05.

## Additional information

**How to cite this article:** Bunda, S. *et al.* Inhibition of SHP2-mediated dephosphorylation of Ras suppresses oncogenesis. *Nat. Commun.* 6:8859 doi: 10.1038/ncomms9859 (2015).

## Supplementary Material

Supplementary Information Supplementary Figures 1-10

## References

[b1] Harvey J. J. An unidentified virus which causes the rapid production of tumours in mice. Nature 204, 1104–1105 (1964). 14243400 10.1038/2041104b0

[b2] Kirsten W. H. & Mayer L. A. Morphologic responses to a murine erythroblastosis virus. J. Natl Cancer Inst. 39, 311–335 (1967). 18623947

[b3] Cox A. D. & Der C. J. Ras history: The saga continues. Small GTPases 1, 2–27 (2010). 21686117 10.4161/sgtp.1.1.12178 PMC3109476

[b4] Barbacid M. ras genes. Annu. Rev. Biochem. 56, 779–827 (1987). 3304147 10.1146/annurev.bi.56.070187.004023

[b5] Wennerberg K., Rossman K. L. & Der C. J. The Ras superfamily at a glance. J. Cell Sci. 118, 843–846 (2005). 15731001 10.1242/jcs.01660

[b6] Campbell S. L., Khosravi-Far R., Rossman K. L., Clark G. J. & Der C. J. Increasing complexity of Ras signaling. Oncogene 17, 1395–1413 (1998). 9779987 10.1038/sj.onc.1202174

[b7] Lowy D. R. & Willumsen B. M. Function and regulation of ras. Annu. Rev. Biochem. 62, 851–891 (1993). 8352603 10.1146/annurev.bi.62.070193.004223

[b8] Milburn M. V. *et al.* Molecular switch for signal transduction: structural differences between active and inactive forms of protooncogenic ras proteins. Science 247, 939–945 (1990). 2406906 10.1126/science.2406906

[b9] Scheffzek K. *et al.* The Ras-RasGAP complex: structural basis for GTPase activation and its loss in oncogenic Ras mutants. Science 277, 333–338 (1997). 9219684 10.1126/science.277.5324.333

[b10] Adari H., Lowy D. R., Willumsen B. M., Der C. J. & McCormick F. Guanosine triphosphatase activating protein (GAP) interacts with the p21 ras effector binding domain. Science 240, 518–521 (1988). 2833817 10.1126/science.2833817

[b11] Mattingly R. R. Activated Ras as a therapeutic target: constraints on directly targeting ras isoforms and wild-type versus mutated proteins. ISRN Oncol. 2013, 536529 (2013). 24294527 10.1155/2013/536529 PMC3833460

[b12] DeClue J. E. *et al.* Abnormal regulation of mammalian p21ras contributes to malignant tumor growth in von Recklinghausen (type 1) neurofibromatosis. Cell 69, 265–273 (1992). 1568246 10.1016/0092-8674(92)90407-4

[b13] Schubbert S. *et al.* Functional analysis of leukemia-associated PTPN11 mutations in primary hematopoietic cells. Blood 106, 311–317 (2005). 15761018 10.1182/blood-2004-11-4207 PMC1895116

[b14] Clark G. J. & Der C. J. Aberrant function of the Ras signal transduction pathway in human breast cancer. Breast Cancer Res. Treat. 35, 133–144 (1995). 7612899 10.1007/BF00694753

[b15] Guha A., Feldkamp M. M., Lau N., Boss G. & Pawson A. Proliferation of human malignant astrocytomas is dependent on Ras activation. Oncogene 15, 2755–2765 (1997). 9419966 10.1038/sj.onc.1201455

[b16] Louis D. N., Holland E. C. & Cairncross J. G. Glioma classification: a molecular reappraisal. Am. J. Pathol. 159, 779–786 (2001). 11549567 10.1016/S0002-9440(10)61750-6 PMC1850454

[b17] Kleihues P. & Ohgaki H. Primary and secondary glioblastomas: from concept to clinical diagnosis. Neuro-oncology 1, 44–51 (1999). 11550301 10.1093/neuonc/1.1.44 PMC1919466

[b18] Holland E. C. Gliomagenesis: genetic alterations and mouse models. Nat. Rev. Genet. 2, 120–129 (2001). 11253051 10.1038/35052535

[b19] Jiang B. H., Aoki M., Zheng J. Z., Li J. & Vogt P. K. Myogenic signaling of phosphatidylinositol 3-kinase requires the serine-threonine kinase Akt/protein kinase B. Proc. Natl Acad. Sci. USA 96, 2077–2081 (1999). 10051597 10.1073/pnas.96.5.2077 PMC26739

[b20] Scott L. M. *et al.* Shp2 protein tyrosine phosphatase inhibitor activity of estramustine phosphate and its triterpenoid analogs. Bioorg. Med. Chem. Lett. 21, 730–733 (2011). 21193311 10.1016/j.bmcl.2010.11.117 PMC3034307

[b21] Bunda S. *et al.* Src promotes GTPase activity of Ras via tyrosine 32 phosphorylation. Proc. Natl Acad. Sci. USA 111, E3785–E3794 (2014). 25157176 10.1073/pnas.1406559111 PMC4246987

[b22] Scott L. M., Lawrence H. R., Sebti S. M., Lawrence N. J. & Wu J. Targeting protein tyrosine phosphatases for anticancer drug discovery. Curr. Pharm. Des. 16, 1843–1862 (2010). 20337577 10.2174/138161210791209027 PMC3076191

[b23] Zhang X. *et al.* Salicylic acid based small molecule inhibitor for the oncogenic Src homology-2 domain containing protein tyrosine phosphatase-2 (SHP2). J. Med. Chem. 53, 2482–2493 (2010). 20170098 10.1021/jm901645u PMC2842125

[b24] Sha F. *et al.* Dissection of the BCR-ABL signaling network using highly specific monobody inhibitors to the SHP2 SH2 domains. Proc. Natl Acad. Sci. USA 110, 14924–14929 (2013). 23980151 10.1073/pnas.1303640110 PMC3773763

[b25] Ding H. *et al.* Astrocyte-specific expression of activated p21-ras results in malignant astrocytoma formation in a transgenic mouse model of human gliomas. Cancer Res. 61, 3826–3836 (2001). 11325859

[b26] Mohi M. G. *et al.* Prognostic, therapeutic, and mechanistic implications of a mouse model of leukemia evoked by Shp2 (PTPN11) mutations. Cancer Cell 7, 179–191 (2005). 15710330 10.1016/j.ccr.2005.01.010

[b27] Matozaki T., Murata Y., Saito Y., Okazawa H. & Ohnishi H. Protein tyrosine phosphatase SHP-2: a proto-oncogene product that promotes Ras activation. Cancer Sci. 100, 1786–1793 (2009). 19622105 10.1111/j.1349-7006.2009.01257.x PMC11158110

[b28] Chan R. J. & Feng G. S. PTPN11 is the first identified proto-oncogene that encodes a tyrosine phosphatase. Blood 109, 862–867 (2007). 17053061 10.1182/blood-2006-07-028829 PMC1785139

[b29] Dance M., Montagner A., Salles J. P., Yart A. & Raynal P. The molecular functions of Shp2 in the Ras/Mitogen-activated protein kinase (ERK1/2) pathway. Cell. Signal. 20, 453–459 (2008). 17993263 10.1016/j.cellsig.2007.10.002

[b30] Ren L. *et al.* Substrate specificity of protein tyrosine phosphatases 1B, RPTPalpha, SHP-1, and SHP-2. Biochemistry 50, 2339–2356 (2011). 21291263 10.1021/bi1014453 PMC3074353

[b31] Huang H. *et al.* A Src family kinase-Shp2 axis controls RUNX1 activity in megakaryocyte and T-lymphocyte differentiation. Genes Dev. 26, 1587–1601 (2012). 22759635 10.1101/gad.192054.112 PMC3404386

[b32] Charpantier E. *et al.* Alpha7 neuronal nicotinic acetylcholine receptors are negatively regulated by tyrosine phosphorylation and Src-family kinases. J. Neurosci. 25, 9836–9849 (2005). 16251431 10.1523/JNEUROSCI.3497-05.2005 PMC6725579

[b33] Huang W. Q. *et al.* Structure, function, and pathogenesis of SHP2 in developmental disorders and tumorigenesis. Curr. Cancer Drug Targets 14, 567–588 (2014). 25039348 10.2174/1568009614666140717105001

[b34] Cunnick J. M., Dorsey J. F., Munoz-Antonia T., Mei L. & Wu J. Requirement of SHP2 binding to Grb2-associated binder-1 for mitogen-activated protein kinase activation in response to lysophosphatidic acid and epidermal growth factor. J. Biol. Chem. 275, 13842–13848 (2000). 10788507 10.1074/jbc.275.18.13842

[b35] Egan S. E. *et al.* Association of Sos Ras exchange protein with Grb2 is implicated in tyrosine kinase signal transduction and transformation. Nature 363, 45–51 (1993). 8479536 10.1038/363045a0

[b36] Montagner A. *et al.* A novel role for Gab1 and SHP2 in epidermal growth factor-induced Ras activation. J. Biol. Chem. 280, 5350–5360 (2005). 15574420 10.1074/jbc.M410012200

[b37] Hanafusa H., Torii S., Yasunaga T., Matsumoto K. & Nishida E. Shp2, an SH2-containing protein-tyrosine phosphatase, positively regulates receptor tyrosine kinase signaling by dephosphorylating and inactivating the inhibitor Sprouty. J. Biol. Chem. 279, 22992–22995 (2004). 15031289 10.1074/jbc.M312498200

[b38] Lee S. H., Schloss D. J., Jarvis L., Krasnow M. A. & Swain J. L. Inhibition of angiogenesis by a mouse sprouty protein. J. Biol. Chem. 276, 4128–4133 (2001). 11053436 10.1074/jbc.M006922200

[b39] Li X., Wheldon L. & Heath J. K. Sprouty: a controversial role in receptor tyrosine kinase signalling pathways. Biochem. Soc. Trans. 31, 1445–1446 (2003). 14641085 10.1042/bst0311445

[b40] Zhang S. Q. *et al.* Shp2 regulates SRC family kinase activity and Ras/Erk activation by controlling Csk recruitment. Mol. Cell 13, 341–355 (2004). 14967142 10.1016/s1097-2765(04)00050-4

[b41] Peyssonnaux C. & Eychene A. The Raf/MEK/ERK pathway: new concepts of activation. Biol. Cell 93, 53–62 (2001). 11730323 10.1016/s0248-4900(01)01125-x

[b42] Tartaglia M. *et al.* Mutations in PTPN11, encoding the protein tyrosine phosphatase SHP-2, cause Noonan syndrome. Nat. Genet. 29, 465–468 (2001). 11704759 10.1038/ng772

[b43] Loh M. L. *et al.* Mutations in PTPN11 implicate the SHP-2 phosphatase in leukemogenesis. Blood 103, 2325–2331 (2004). 14644997 10.1182/blood-2003-09-3287

[b44] Tartaglia M. *et al.* Somatic PTPN11 mutations in childhood acute myeloid leukaemia. Br. J. Haematol. 129, 333–339 (2005). 15842656 10.1111/j.1365-2141.2005.05457.x

[b45] Loh M. L. *et al.* PTPN11 mutations in pediatric patients with acute myeloid leukemia: results from the Children's Cancer Group. Leukemia 18, 1831–1834 (2004). 15385933 10.1038/sj.leu.2403492

[b46] Tartaglia M. *et al.* Somatic mutations in PTPN11 in juvenile myelomonocytic leukemia, myelodysplastic syndromes and acute myeloid leukemia. Nat. Genet. 34, 148–150 (2003). 12717436 10.1038/ng1156

[b47] Yamamoto T. *et al.* PTPN11, RAS and FLT3 mutations in childhood acute lymphoblastic leukemia. Leuk. Res. 30, 1085–1089 (2006). 16533526 10.1016/j.leukres.2006.02.004

[b48] Aceto N. *et al.* Tyrosine phosphatase SHP2 promotes breast cancer progression and maintains tumor-initiating cells via activation of key transcription factors and a positive feedback signaling loop. Nat. Med. 18, 529–537 (2012). 22388088 10.1038/nm.2645

[b49] Rankin J., Short J., Turnpenny P., Castle B. & Hanemann C. O. Medulloblastoma in a patient with the PTPN11 p.Thr468Met mutation. Am. J. Med. Genet. A 161A, 2027–2029 (2013). 23813970 10.1002/ajmg.a.36005

[b50] Bentires-Alj M. *et al.* Activating mutations of the noonan syndrome-associated SHP2/PTPN11 gene in human solid tumors and adult acute myelogenous leukemia. Cancer Res. 64, 8816–8820 (2004). 15604238 10.1158/0008-5472.CAN-04-1923

[b51] Cotton J. L. & Williams R. G. Noonan syndrome and neuroblastoma. Arch. Pediatr. Adolesc. Med. 149, 1280–1281 (1995). 7581766 10.1001/archpedi.1995.02170240098019

[b52] Lopez-Miranda B., Westra S. J., Yazdani S. & Boechat M. I. Noonan syndrome associated with neuroblastoma: a case report. Pediatr. Radiol. 27, 324–326 (1997). 9162899 10.1007/s002470050140

[b53] Martinelli S. *et al.* Activating PTPN11 mutations play a minor role in pediatric and adult solid tumors. Cancer Genet. Cytogenet. 166, 124–129 (2006). 16631468 10.1016/j.cancergencyto.2005.10.003

[b54] Miyamoto D. *et al.* Isolation of a distinct class of gain-of-function SHP-2 mutants with oncogenic RAS-like transforming activity from solid tumors. Oncogene 27, 3508–3515 (2008). 18223690 10.1038/sj.onc.1211019

[b55] Sturla L. M. *et al.* Src homology domain-containing phosphatase 2 suppresses cellular senescence in glioblastoma. Br. J. Cancer 105, 1235–1243 (2011). 21934682 10.1038/bjc.2011.345 PMC3208488

[b56] Janzarik W. G. *et al.* Further evidence for a somatic KRAS mutation in a pilocytic astrocytoma. Neuropediatrics 38, 61–63 (2007). 17712732 10.1055/s-2007-984451

[b57] Cancer Genome Atlas Research Network. Comprehensive genomic characterization defines human glioblastoma genes and core pathways. Nature 455, 1061–1068 (2008). 18772890 10.1038/nature07385 PMC2671642

[b58] Liu K. W. *et al.* SHP-2/PTPN11 mediates gliomagenesis driven by PDGFRA and INK4A/ARF aberrations in mice and humans. J. Clin. Invest. 121, 905–917 (2011). 21393858 10.1172/JCI43690 PMC3049395

[b59] Feng H. *et al.* Dynamin 2 mediates PDGFRalpha-SHP-2-promoted glioblastoma growth and invasion. Oncogene 31, 2691–2702 (2012). 21996738 10.1038/onc.2011.436 PMC3262067

[b60] Zhan Y., Counelis G. J. & O'Rourke D. M. The protein tyrosine phosphatase SHP-2 is required for EGFRvIII oncogenic transformation in human glioblastoma cells. Exp. Cell Res. 315, 2343–2357 (2009). 19427850 10.1016/j.yexcr.2009.05.001 PMC2724964

[b61] Wei J. *et al.* Glioma-associated cancer-initiating cells induce immunosuppression. Clinical Cancer Res. 16, 461–473 (2010). 20068105 10.1158/1078-0432.CCR-09-1983 PMC2943842

[b62] Chung C. *et al.* Imaging biomarker dynamics in an intracranial murine glioma study of radiation and antiangiogenic therapy. Int. J. Radiat Oncol. Biol. Phys. 85, 805–812 (2013). 22929856 10.1016/j.ijrobp.2012.07.005 PMC8366682

[b63] Ohh M. *et al.* Ubiquitination of hypoxia-inducible factor requires direct binding to the beta-domain of the von Hippel-Lindau protein. Nat. Cell Biol. 2, 423–427 (2000). 10878807 10.1038/35017054

