# Supplementary material for: Inhibition of SHP2-mediated dephosphorylation of Ras suppresses oncogenesis
Source: Nat Commun. 2015 Nov 30;6:8859. doi: 10.1038/ncomms9859 (PMC4674766; doi:10.1038/ncomms9859)
Supplement: Supplementary Information — Supplementary Figures 1-10 [file ncomms9859-s1.pdf]

SUPPLEMENTARY INFORMATION

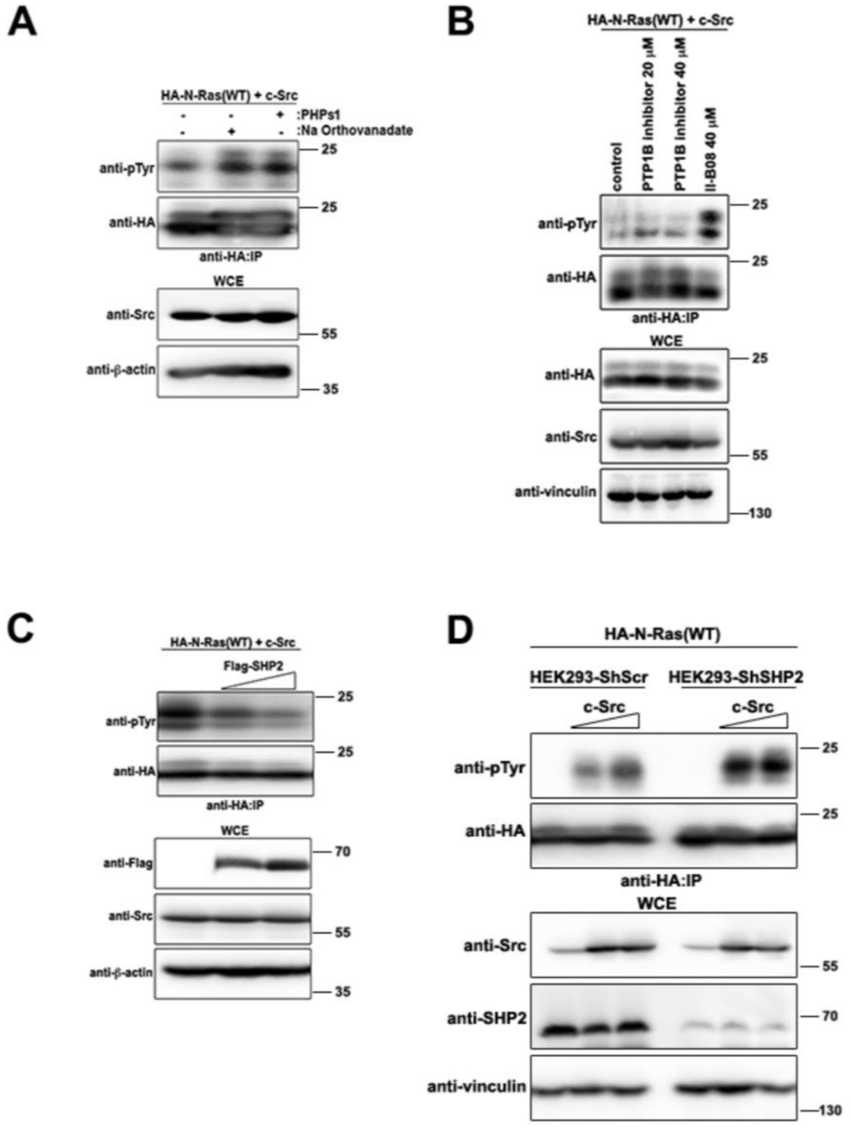

Supplementary Figure 1. SHP2 dephosphorylates Ras. (A) HEK293 cells transfected with indicated plasmids were treated with (+) or without (-) PHPs1 or sodium orthovanadate, lysed, immunoprecipitated using anti-HA antibody, and immunoblotted with indicated antibodies. (B) HEK293 cells transfected with indicated plasmids were treated with (+) or without (-) increasing concentrations of PTP1B or II-B08, lysed, immunoprecipitated using anti-HA antibody, and immunoblotted with indicated antibodies. (C) HEK293 cells transfected with indicated plasmids were lysed, immunoprecipitated using anti-HA antibody, and immunoblotted with the indicated antibodies. (D) HEK293-ShScr or -ShSHP2 cells transfected with indicated plasmids were lysed, immunoprecipitated using anti-HA antibody, and immunoblotted with the indicated antibodies. The immunoblot data are representative of at least three separate experiments. WCE: whole cell extract; IP: immunoprecipitation; PD: pull down.

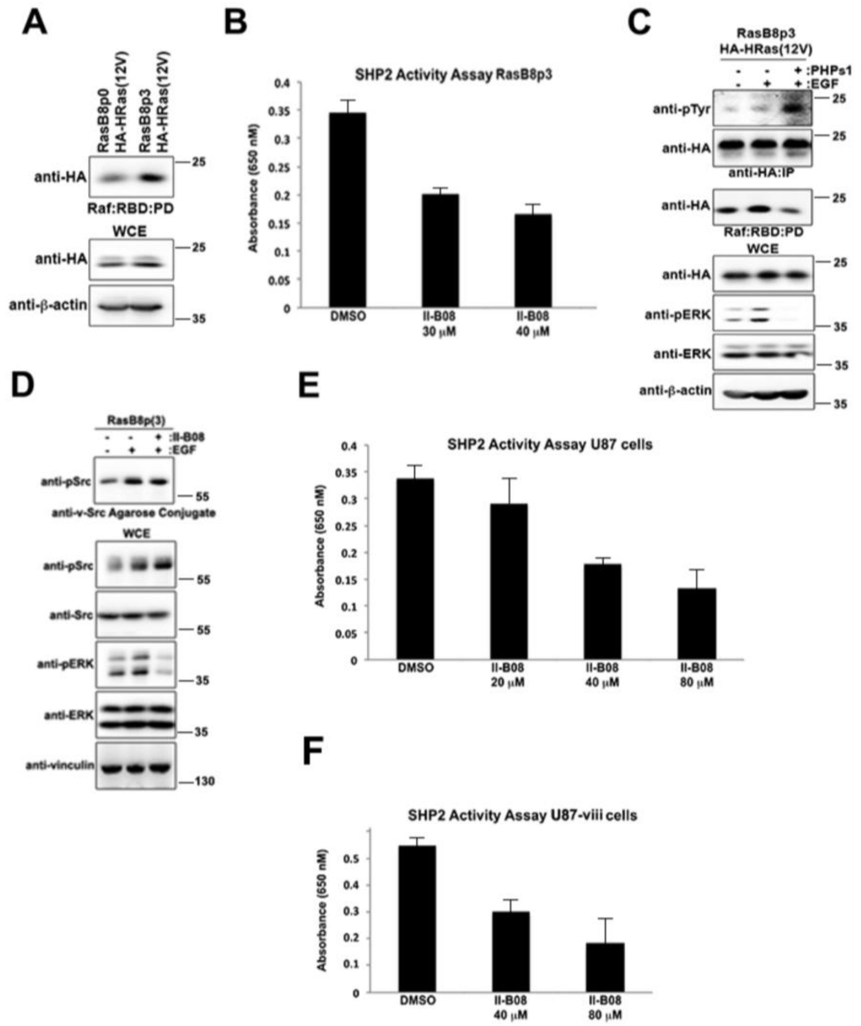

Supplementary Figure 2. II-B08 inactivates endogenous SHP2 activity in RasB8p3, U87 and U87viii astrocytes. (A) RasB8p0 or RasB8p3 astrocytes were lysed, Raf:RBD pull down assay was performed and immunoblotted with the indicated antibodies. (B) RasB8p3 astrocytes were treated with or without increasing concentrations of II-B08, lysed and SHP2 activity assay was performed in triplicates at least three independent times. (C) RasB8p3 astrocytes were serum starved and treated with (+) or without (-) PHPs1 in the presence (+) or absence (-) of EGF. Cells were lysed, Raf:RBD pull down assay or immunoprecipitation using anti-HA antibody was performed and immunoblotted with the indicated antibodies. (D) RasB8p3 astrocytes were treated with (+) or without (-) II-B08 in the presence (+) or absence (-) of EGF. Cells were lysed, immunoprecipitated with anti-v-Src agarose-conjugated antibody, and immunoblotted with the indicated antibodies. (E and F) U87 or U87viii astrocytes were treated with or without increasing concentrations of II-B08, lysed and SHP2 activity assay was performed in triplicates at least three independent times. The immunoblot data are representative of at least three separate experiments. WCE: whole cell extract; IP: immunoprecipitation; PD: pull down.

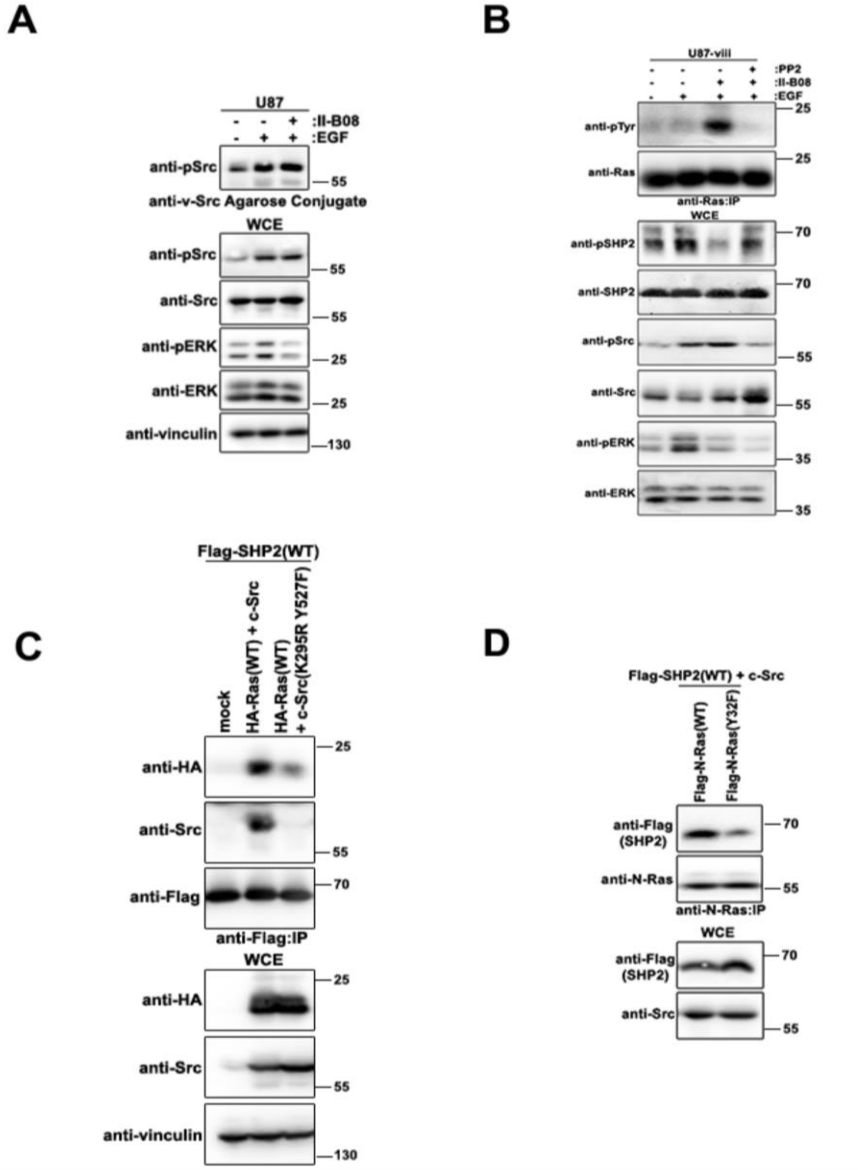

Supplementary Figure 3. SHP2 dephosphorylates Src-induced tyrosyl phosphorylated Ras. (A) U87 astrocytes were treated with (+) or without (-) II-B08 in the presence (+) or absence (-) of EGF. Cells were lysed, immunoprecipitated with anti-v-Src agarose-conjugated antibody, and immunoblotted with the indicated antibodies. (B) U87viii astrocytes serum starved and pretreated with (+) or without (-) PP2 or II-B08 in the presence (-) or absence (-) of EGF were lysed, immunoprecipitated with anti-Ras antibody, and immunoblotted with the indicated antibodies. (C and D) HEK293 cells transfected with the indicated plasmids were lysed, immunoprecipitated with anti-Flag or anti-Ras antibody, and immunoblotted with the indicated antibodies. The immunoblot data are representative of at least three separate experiments. WCE: whole cell extract; IP: immunoprecipitation.

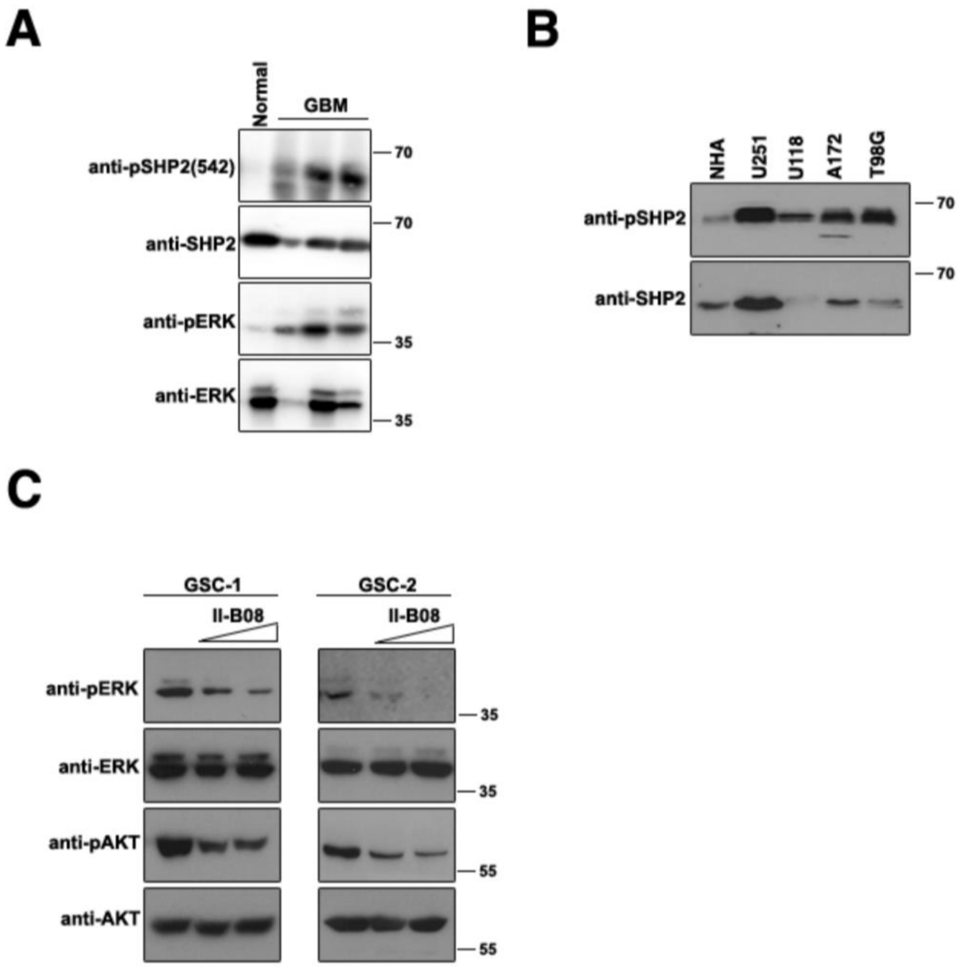

Supplementary Figure 4. Human primary GBM tumour samples, GBM cell lines and glioma stem cell (GSC) neurosphere cultures show elevated levels of SHP2 phosphorylation. (A) Patient derived GBM samples or normal human tissues were lysed and immunoblotted with the indicated antibodies. (B) Indicated human GBM cell lines or NHA were lysed and immunoblotted with the indicated antibodies. (C) GSC neurosphere cultures were treated with increasing concentrations of II-B08, lysed and immunoblotted with the indicated antibodies. The immunoblot data are representative of at least three separate experiments.

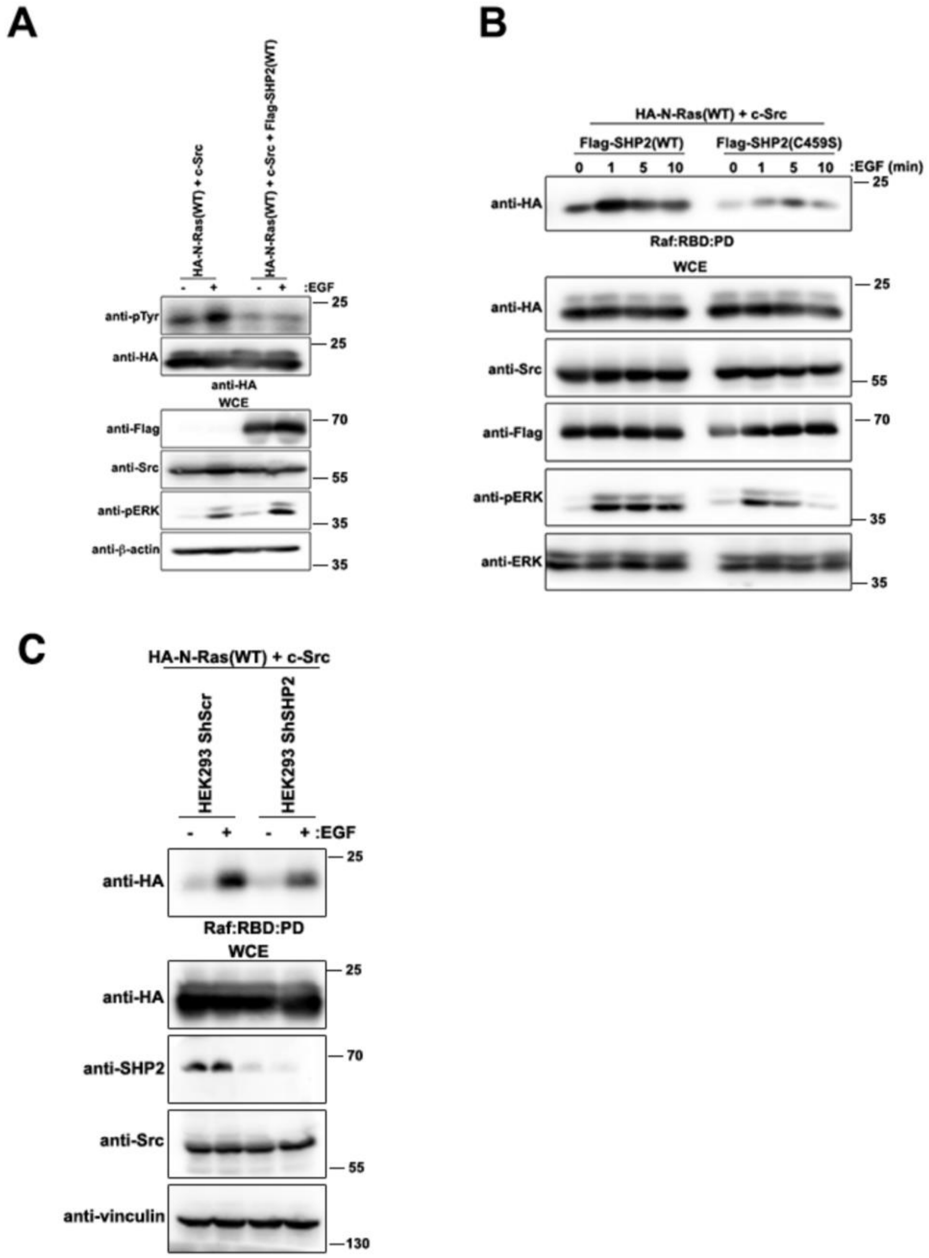

Supplementary Figure 5. SHP2 dephosphorylates Ras to activate downstream ERK signalling. HEK293 cells transfected with the indicated combination of plasmids were lysed, immunoprecipitated with anti-HA antibody (A) or pulled down using Raf:RBD beads (B) and immunoblotted with the indicated antibodies. (C) HEK293-ShScr or -ShSHP2 cells were transfected with indicated combination of plasmids and treated with (+) or without (-) EGF, lysed, pulled down using Raf:RBD beads, and immunoblotted with the indicated antibodies. The immunoblot data are representative of at least three separate experiments. WCE: whole cell extract; IP: immunoprecipitation; PD: pull down.

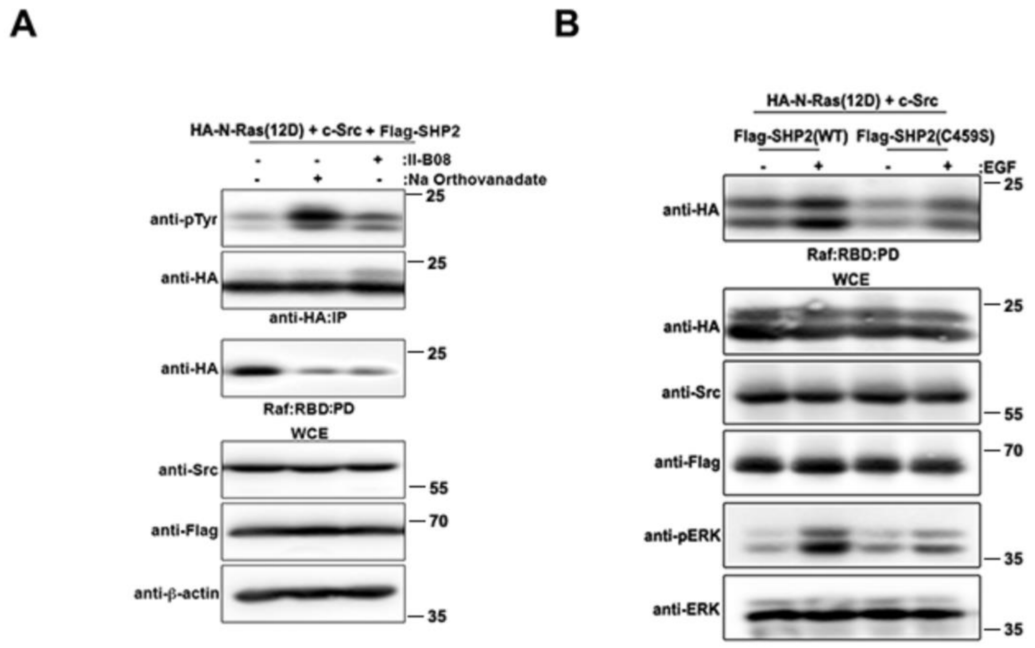

Supplementary Figure 6. SHP2 inhibition inactivates Ras and its downstream signaling. (A) HEK293 cells transfected with the indicated plasmids were treated with (+) or without (-) II-B08 or sodium orthovanadate were lysed, immunoprecipitated with anti-HA antibody or pulled down using Raf:RBD beads, and immunoblotted with the indicated antibodies. (B) HEK293 cells transfected with the indicated plasmids were lysed, pulled down using Raf:RBD beads and immunoblotted with the indicated antibodies. The immunoblot data are representative of at least three separate experiments. WCE: whole cell extract; IP: immunoprecipitation; PD: pull down.

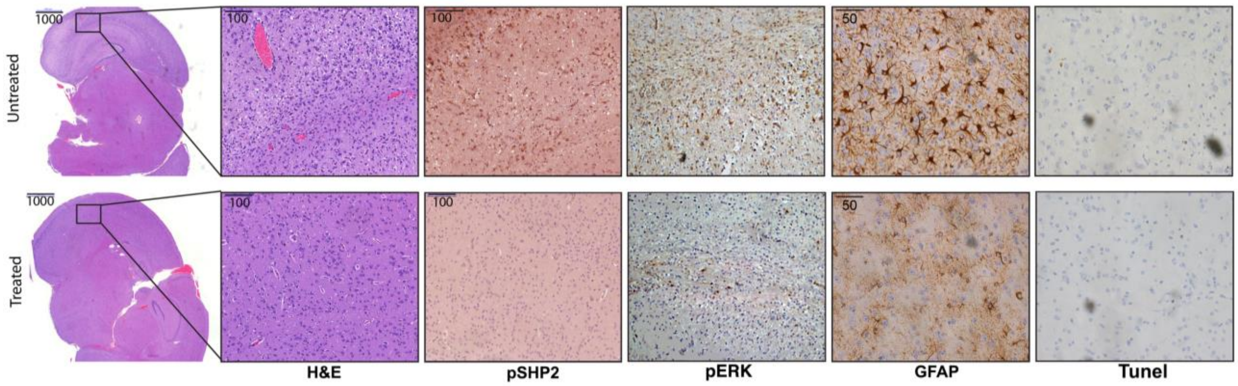

Supplementary Figure 7. RasB8 mice treated with II-B08 are less tumourigenic. H&E staining and IHC of pSHP2, pERK, GFAP, and TUNEL demonstrate a reduction in the number of nuclei and as such the proliferative index of the tissue. In the brains where there is marked SHP2 inhibition, there is a reduction in GFAP expression suggesting a less astrocytic nature tumour in the treated mice. Apoptosis levels are relatively unchanged. Images are representative from 6 untreated or 6 treated 6 weak-old mice with II-B08.

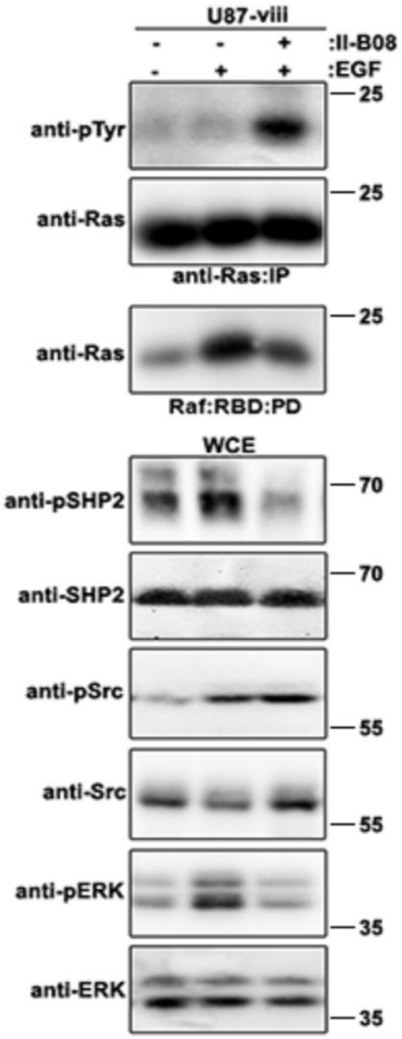

Supplementary Figure 8. SHP2 inhibition inactivates Ras activity in U87viii astrocytes. U87viii astrocytes serum starved and pretreated with (+) or without (-) II-B08 following treatment with (+) or without (-) EGF were lysed, pulled down using Raf:RBD conjugated beads and immunoblotted with the indicated antibodies. The immunoblot data are representative of at least three separate experiments. PD: pull down.

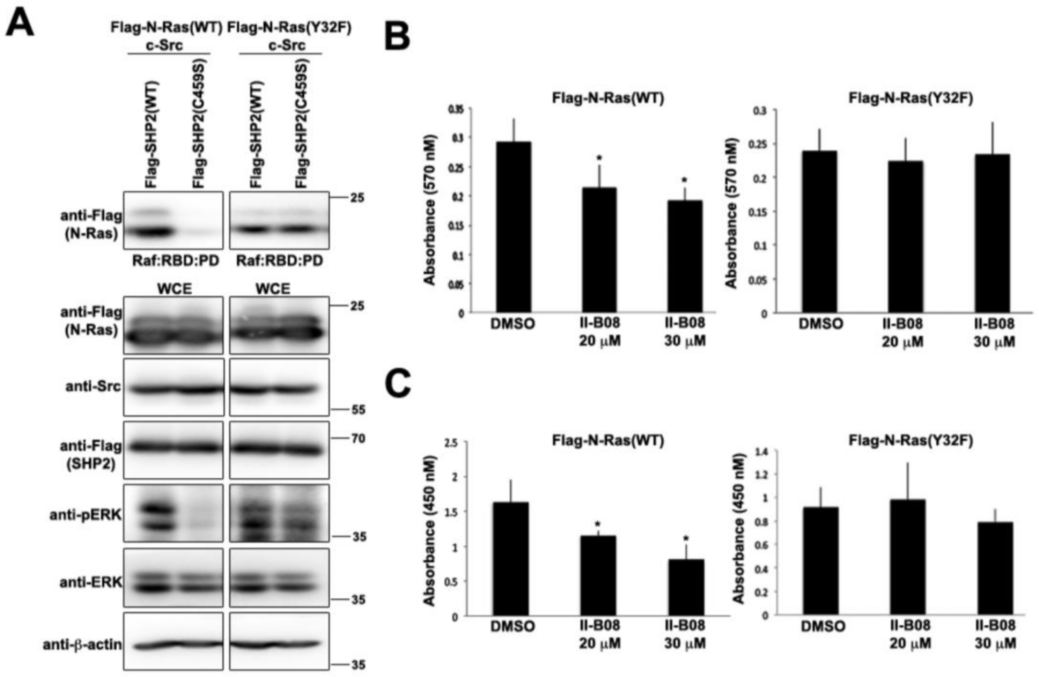

Supplementary Figure 9. SHP2 inhibition does not markedly affect proliferation of Flag-N-RasY32F-expressing HEK293 cells. (A) HEK293 cells transfected with the indicated plasmids were lysed, Raf:RBD pull down assay performed and immunoblotted with the indicated antibodies. Equal number of HEK293 cells transfected with indicated plasmids were plated in 96-well plates in sextuplicate, treated with or without (DMSO) increasing concentration of II-B08 for 18 h, and alamar blue (B) or BrdU (C) assay was then performed. Data represent mean ± s.e.m. of three independent experiments performed in sextuplicates. *$P < 0.05$ Student's t test compared to DMSO control.

Fig 1A

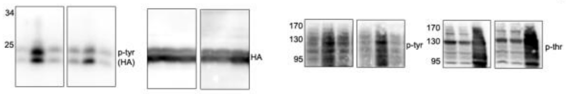

Fig 1B

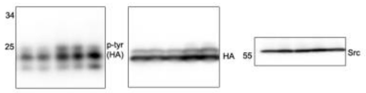

Fig 1C

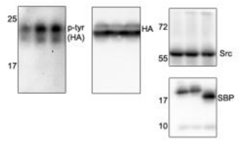

Fig 1D

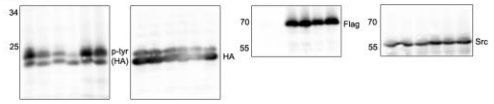

Fig 1E

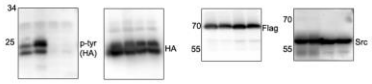

Fig 1F

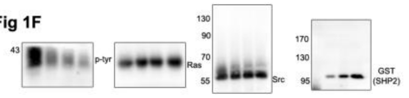

Fig 2A

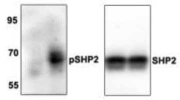

Fig 2B

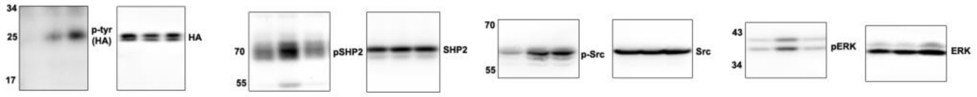

Fig 2C

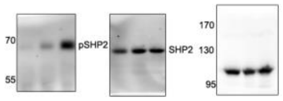

Fig 2D

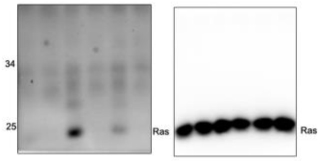

Fig 2E

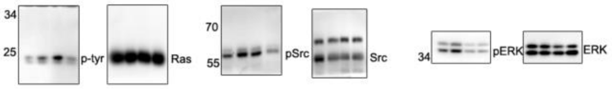

Fig 2F

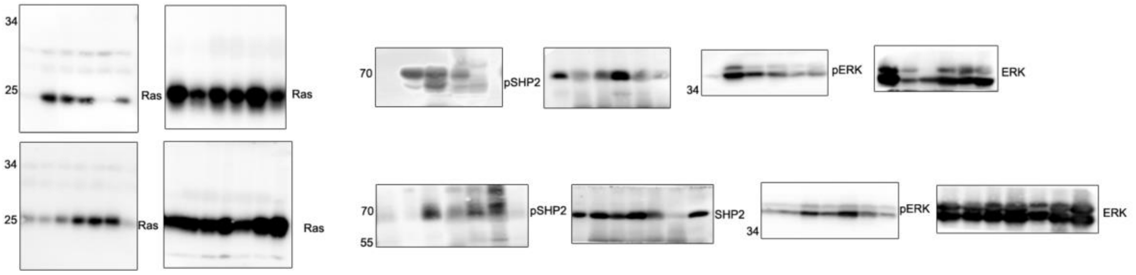

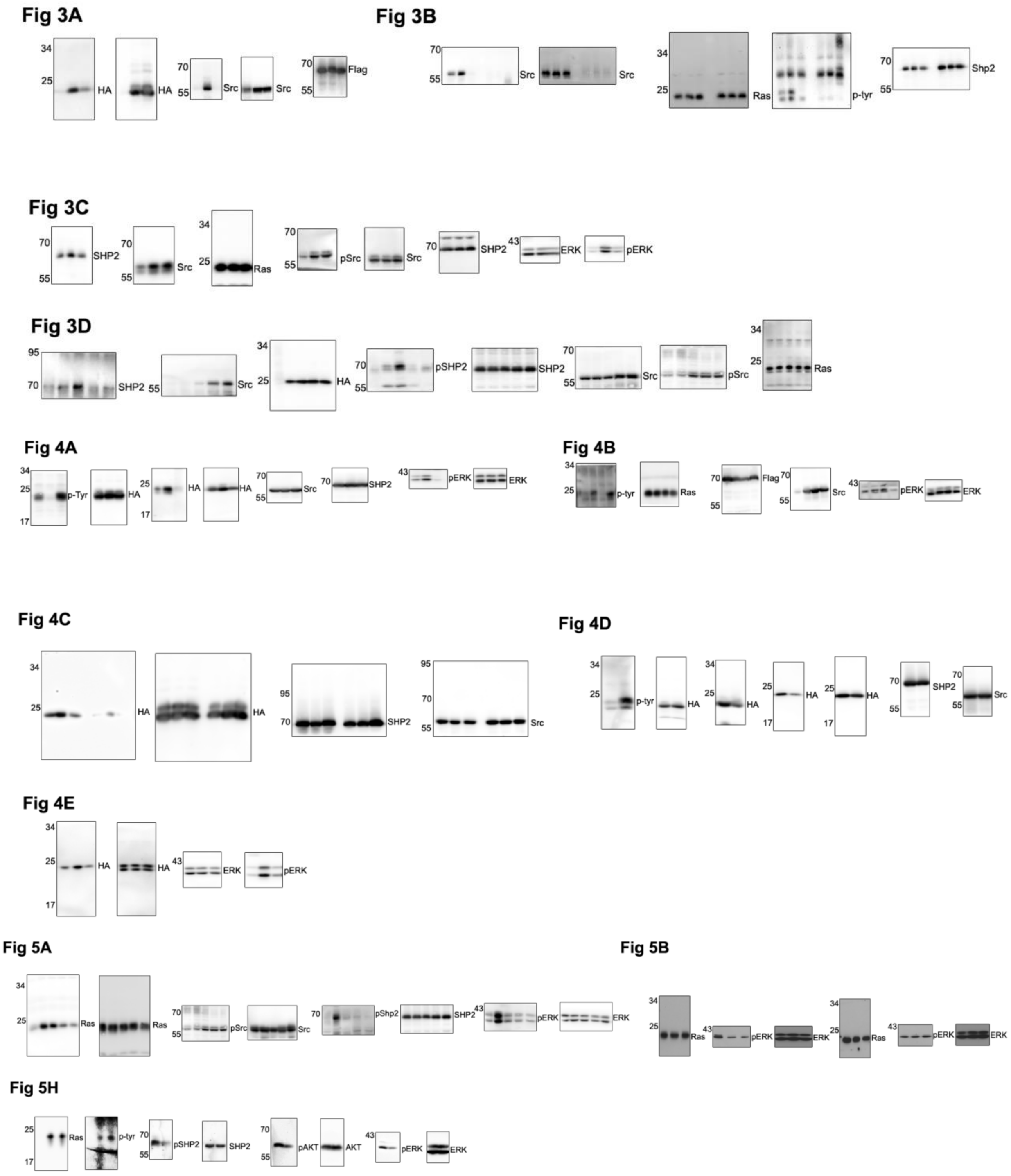

Supplementary Figure 10: Uncropped blots of main figures.
